# Neural dynamics of delayed feedback in robot teleoperation: insights from fNIRS analysis

**DOI:** 10.3389/fnhum.2024.1338453

**Published:** 2024-06-17

**Authors:** Tianyu Zhou, Yang Ye, Qi Zhu, William Vann, Jing Du

**Affiliations:** ^1^The Informatics, Cobots and Intelligent Construction (ICIC) Lab, Department of Civil and Coastal Engineering, University of Florida, Gainesville, FL, United States; ^2^Communications Technology Laboratory, Public Safety Communications Research Division, Advanced Communications Research Group, National Institute of Standards and Technology, Boulder, CO, United States

**Keywords:** robot teleoperation, functional Near-Infrared Spectroscopy (fNIRS), sensory feedback delays, haptic feedback, cortical activation in teleoperation

## Abstract

**Introduction:**

As robot teleoperation increasingly becomes integral in executing tasks in distant, hazardous, or inaccessible environments, operational delays remain a significant obstacle. These delays, inherent in signal transmission and processing, adversely affect operator performance, particularly in tasks requiring precision and timeliness. While current research has made strides in mitigating these delays through advanced control strategies and training methods, a crucial gap persists in understanding the neurofunctional impacts of these delays and the efficacy of countermeasures from a cognitive perspective.

**Methods:**

This study addresses the gap by leveraging functional Near-Infrared Spectroscopy (fNIRS) to examine the neurofunctional implications of simulated haptic feedback on cognitive activity and motor coordination under delayed conditions. In a human-subject experiment (*N* = 41), sensory feedback was manipulated to observe its influences on various brain regions of interest (ROIs) during teleoperation tasks. The fNIRS data provided a detailed assessment of cerebral activity, particularly in ROIs implicated in time perception and the execution of precise movements.

**Results:**

Our results reveal that the anchoring condition, which provided immediate simulated haptic feedback with a delayed visual cue, significantly optimized neural functions related to time perception and motor coordination. This condition also improved motor performance compared to the asynchronous condition, where visual and haptic feedback were misaligned.

**Discussion:**

These findings provide empirical evidence about the neurofunctional basis of the enhanced motor performance with simulated synthetic force feedback in the presence of teleoperation delays. The study highlights the potential for immediate haptic feedback to mitigate the adverse effects of operational delays, thereby improving the efficacy of teleoperation in critical applications.

## 1 Introduction

Robot teleoperation enables human operators to command and control robots in distant, hazardous, or inaccessible environments (Senft et al., 2021). This ability expands the range of feasible applications, such as deep-sea exploration, space missions, and hazardous material handling, allowing for complex tasks to be conducted beyond the conventional spatial limitations imposed between the human operator and the robot (Zhou et al., 2023). However, the potential of teleoperation is often undermined by operational delays due to physical constraints like signal transmission distances and processing limits, resulting in latency that affects situational awareness, control precision, and task performance (Kluge et al., 2013; Wenhao et al., 2017; Payra et al., 2020). Such delays increase cognitive workload, error potential, and challenge the efficiency and effectiveness of teleoperation (Orlosky et al., 2018; Kim E. et al., 2021).

In order to mitigate the implications of inevitable delays in robot teleoperation, literature has presented a variety of technical or behavioral countermeasures (Farajiparvar et al., 2020). Prominent among these countermeasures include supervisory controls (Manoharan and Ponraj, 2019), predictive controls (Uddin and Ryu, 2016), diversified of interaction modalities (Magrini et al., 2020), and intensive trainings for developing adaptive manipulative tactics such as the “move and wait” strategy (Hokayem and Spong, 2006). These countermeasures aim at optimizing the reactive actions based on the predicted delay patterns (Zhu et al., 2023), or improve human responses while repetitive training (Pervez et al., 2019). Nevertheless, these existing methods are less effective when delay patterns are less clear, or when training is limited such as in emergent scenarios. To prepare for more extreme conditions of delayed teleoperation, we have proposed an innovative approach to sensory manipulation. By utilizing a physics engine, we simulate synthetic force feedback in anticipation of the actual haptic signal data (Du et al., 2023). This method creates a more intuitive and responsive teleoperation experience, even when communication delays change. The simulated feedback is designed to approximate the real physical interactions that the robot would experience, providing the operator with a preemptive sense of the forces involved in the task. In our pilot test we have found that this sensory manipulation method could significantly improve the operator's perception and control, thereby reducing the adverse effects of the inherent delays in robot teleoperation.

However, we noticed a knowledge gap in terms of the neurofunctional underpinnings of sensory manipulation or other similar approaches as countermeasures to teleportation delays. While existing studies have examined the implications of teleoperation delays and corresponding mitigation strategies on motor performance, or self-assessment of perception and cognitive status, there remains a significant gap in understanding how these strategies affect neural functions, particularly those related to time perception and motor coordination. The existing literature largely neglects the neural underpinnings that could play a crucial role in determining the efficacy of teleoperated manipulations. Specifically, there is a scarcity of evidence on how synthetic, simulated haptic feedback influences these neural processes. This omission is critical as understanding the neurofunctional impacts of sensory manipulation could provide deeper insights into the mechanisms through which these strategies improve teleoperation performance.

In addressing the challenges posed by teleoperation delays, it's crucial to understand their impact on neural functions and motor coordination, which are essential for precise task execution. Research highlights that the basal ganglia and supplementary motor areas play pivotal roles in timing and motor coordination, directly influencing teleoperated task performance under latency conditions (Halsband et al., 1993; Merchant et al., 2013). Moreover, advancements in neuroimaging techniques, particularly functional Near-Infrared Spectroscopy (fNIRS), have provided insights into how these delays impact the prefrontal and motor cortices, areas crucial for decision-making and movement execution (Sanes and Donoghue, 2000; Zimeo Morais et al., 2018). Our study leverages fNIRS technology to enhance teleoperation system design and training, aiming to improve operator performance and mitigate the challenges of delayed feedback.

In designing the conditions for this study, we focused on realistic teleoperation scenarios characterized by long-distance communication where visual and haptic data transmission times differ significantly. Visual data, often large in size such as a frame of 1080p video, tends to incur longer transmission delays compared to haptic data, which typically consists of smaller packets (e.g., six floats data for force and torque). This difference is caused by inherent differences in data size and transmission requirements, leading us to hypothesize that visual delays would generally be greater than haptic delays in real-world teleoperation applications. Our study aimed to explore how these common delay scenarios affect both neurofunctional responses and task performance in teleoperation, providing insights that could guide the optimization of teleoperation systems, particularly in fields requiring high precision and rapid feedback.

**Figure 1:**
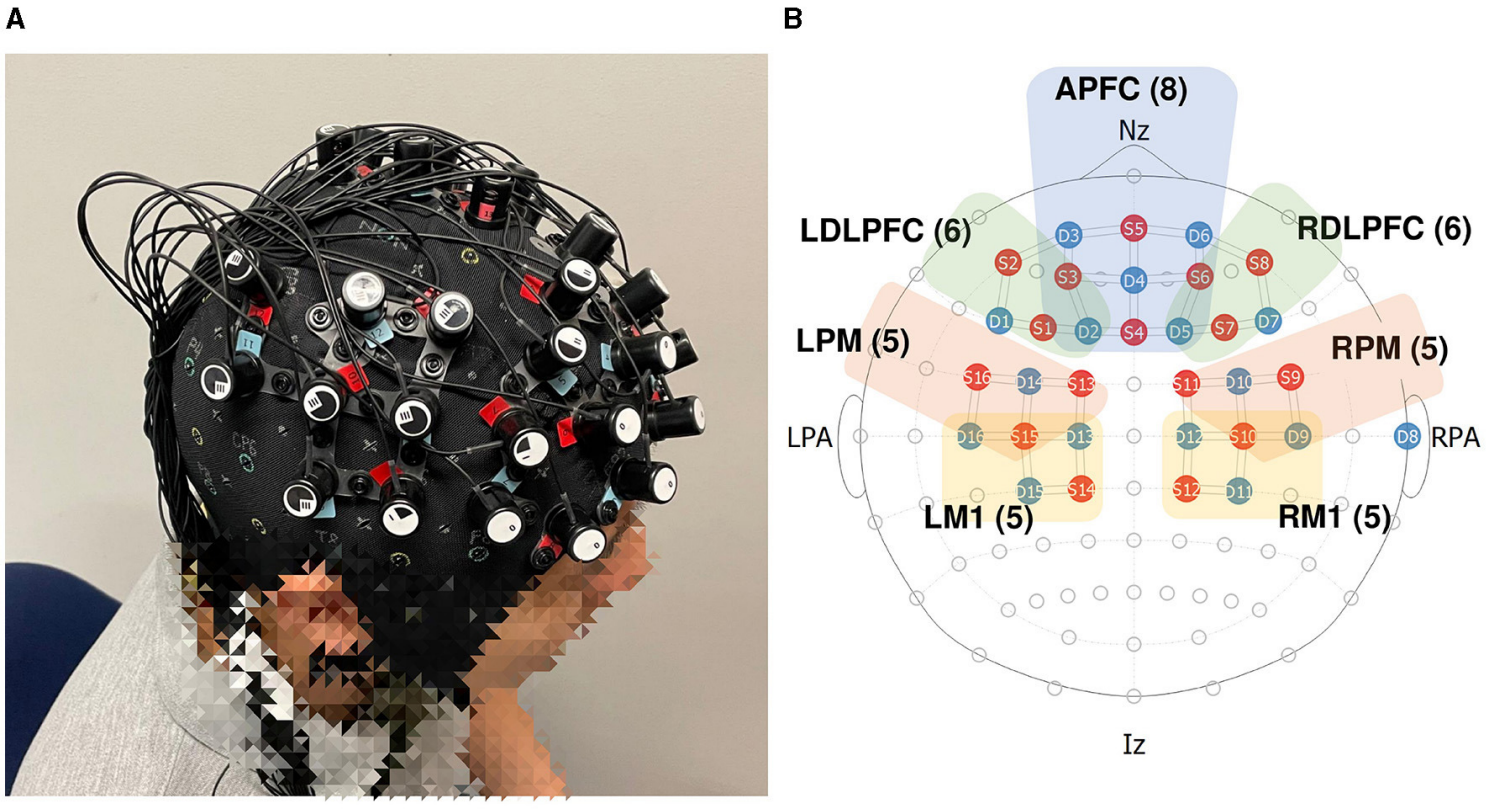
fNIRS layout setting. **(A)** Wearing of real fNIRS; **(B)** region of interest (ROI).

The objective of this paper is to address this knowledge gap by exploring the neurofunctional implications of synthetic haptic feedback in delayed robot teleoperation. To this end, we have conducted a human-subject experiment (*N* = 41), utilizing fNIRS to monitor neural activity. Our study concentrated on analyzing data from several key brain regions relevant to robot teleoperation: the anterior prefrontal cortex (APFC), left and right dorsolateral prefrontal cortex (LDLPFC and RDLPFC), left and right premotor cortex (LM1 and RM1), and left and right primary motor cortex (LPM and RPM) as illustrated in Figure 1. The specific channels designated for each ROI are below:

**APFC (8):** S5-D3, S5-D6, S5-D4, S3-D4, S6-D4, S4-D4, S4-D2, S4-D5.

**LDLPFC (6):** S2-D3, S2-D1, S1-D1, S1-D2, S3-D3, S3-D2.

**RDLPFC (6):** S8-D6, S8-D7, S7-D7, S7-D5, S6-D6, S6-D5.

**LPM (5):** S16-D16, S16-D14, S15-D14, S13-D14, S13-D13.

**RPM (5):** S9-D9, S9-D10, S10-D10, S11-D10, S11-D12.

**LM1 (5):** S15-D16, S15-D15, S15-D13, S14-D15, S14-D13.

**RM1 (5):** S10-D9, S10-D11, S10-D12, S12-D11, S12-D12.

Each selected region plays a crucial role in teleoperation: the APFC is involved in executive functions and complex problem-solving (Euston et al., 2012; Carlén, 2017), the LDLPFC and RDLPFC in working memory and decision-making processes (Philiastides et al., 2011; Kim K. et al., 2021; Martin et al., 2024), the LM1 and RM1 in movement planning (Hoshi and Tanji, 2000; Garbarini et al., 2019; Gale et al., 2021), and the LPM and RPM are involved in the execution of movements (Schnitzler et al., 1997; Solopchuk et al., 2016). Notably, the APFC, LDLPFC, and RDLPFC also contribute to the perception of time, a cognitive function that becomes especially important in the context of feedback delays where the brain must reconcile the discrepancy between expected and actual sensory inputs (Wei-Cong et al., 2015; Coull et al., 2016).

This study primarily aims to provide empirical evidence on how adjustments to force feedback timing influence neural functions related to time perception and motor coordination, thereby offering a neuroscientific perspective on the effectiveness of sensory manipulation in enhancing teleoperated task performance. While we also consider the role of visual feedback, our focus is on filling the knowledge gap regarding force feedback's unique and interactive effects with visual cues. This approach enables us to explore how both types of feedback jointly influence human performance and brain activity in teleoperation, particularly under conditions of latency. The remainder of the paper introduces the relevant body of literature, details the design of our experiment, and discusses the key findings, emphasizing the impact of optimized force feedback in complex teleoperation scenarios.

## 2 Literature review

### 2.1 Neural functions in temporal motor tasks in teleoperation

Understanding how teleoperation delays impact neural functions, particularly time perception and motor coordination, is crucial for addressing the challenges in robot teleoperation. The principles of optimality in sensorimotor control, which suggest that the brain minimizes costs like effort or error despite feedback delays, are crucial for navigating teleoperation complexities (Li et al., 2022; Ijspeert and Daley, 2023). Understanding how these optimization strategies are employed can provide deeper insights into the adaptive mechanisms in teleoperation. The first noticeable function is the time perception ability. The role of time perception in tasks requiring precise timing, such as in surgical procedures or precision engineering, is critical. The integration of optimal feedback control (OFC) mechanisms is vital for maintaining precision in teleoperated tasks, particularly where time perception and motor synchronization are challenged by latency (Sheng et al., 2023). OFC principles can explain how individuals adapt their sensorimotor behaviors to maintain efficiency and accuracy, even when the timing of feedback is altered (Razavian et al., 2023). Studies like Block and Zakay (1996) have explored the subjective nature of time perception, indicating its susceptibility to various factors, including task complexity and attentional resources. Ivry and Spencer (2004) further emphasize the intrinsic link between time perception and motor functions, particularly in tasks requiring synchronization and rhythm. In teleoperation, particularly in precision-demanding tasks like surgical operations or complex machinery control, the synchronization of motor responses with perceived time is critical. Altered time perception due to latency, as demonstrated in studies such as Merchant et al. (2013), can significantly impact the accuracy of these tasks. This highlights a crucial area for teleoperation systems design: minimizing latency effects to improve time perception accuracy and thus task performance.

Research has identified that the basal ganglia are central to timing and time perception, crucial for teleoperation tasks that require millisecond to second precision (Merchant et al., 2013; McElvain et al., 2021; Baladron et al., 2023). Additionally, the supplementary motor area (SMA) and pre-SMA are involved in integrating temporal and motor information, essential for planning and timing movements (Halsband et al., 1993; Mondok and Wiener, 2023). Furthermore, the dorsolateral prefrontal cortex (DLPFC) is implicated in the cognitive aspects of time perception (Wei-Cong et al., 2015). Studies by Yin et al. (2019) and Onoe et al. (2001) suggest the DLPFC's role in temporal discrimination and the cognitive control of time estimation, crucial for adjusting to delays in teleoperation. In the context of teleoperation, where operators need to integrate temporal judgments with motor coordination and decision-making, the role of the DLPFC could be significant. It may contribute to how operators perceive and adjust to delays, particularly in tasks that require them to maintain and manipulate temporal information over short periods.

Motor coordination, crucial for executing complex teleoperated tasks, depends significantly on the quality and timeliness of sensory feedback, with studies emphasizing the critical role of accurate haptic feedback (Ankarali et al., 2014). Further, research by Tin and Poon (2005) on internal models in sensorimotor integration suggests that delays in feedback can disrupt these internal models, leading to a misalignment between intended and executed actions. The impact of this misalignment in high-precision tasks, as highlighted in the work of Jones and Kandathil (2018), underscores the necessity for real-time or predictive sensory inputs in teleoperation. Literature has provided solid evidence about the neurofunctional ROIs related to the motor coordination. For example, the primary motor cortex, as shown by Hari et al. (1998) and Seghezzi and Zapparoli (2020), is pivotal not only in movement execution but also in motor planning, adapting strategies in dynamic environments typical of teleoperation. Scott (2012) and Albert and Shadmehr (2016) further illustrate its role in encoding movement parameters and adapting motor plans in response to feedback, crucial under teleoperation delays. Complementing this, the cerebellum, highlighted in studies by Fautrelle et al. (2011) and Johnson et al. (2019), plays an essential role in fine-tuning movements and error correction, ensuring smooth and coordinated motor output. Its involvement in predictive motor control, as noted by Witney et al. (1999) and Zhu et al. (2023), is particularly relevant for anticipating and compensating for communication delays in teleoperation. The synergy between the primary motor cortex and the cerebellum, as discussed by Galea et al. (2011), is fundamental in maintaining precision and control, adapting, and compensating for the delayed feedback inherent in teleoperated tasks.

Following this discussion, it is crucial to incorporate recent insights into the optimality principles of sensorimotor control, which emphasize the role of OFC in achieving efficient and accurate motor responses. The OFC framework suggests that the central nervous system optimally integrates sensory feedback with predictions of future states to minimize the variance of movement errors (Todorov and Jordan, 2002). This principle is particularly significant in teleoperation, where feedback delays can disrupt the sensory-motor loop. Incorporating OFC principles can lead to the development of teleoperated systems that better compensate for these delays by adjusting the control algorithms to anticipate and mitigate the impact of latency on motor accuracy (Mitrovic et al., 2010; Zhu et al., 2023). Studies such as Franklin and Wolpert (2011) have demonstrated that applying OFC in robotic systems enables more adaptive and resilient responses to unexpected changes or errors in movement execution, enhancing the overall effectiveness of teleoperated tasks (Zhang et al., 2023).

It is also noted that investigating how simulated feedback influences specific brain regions can provide critical insights into the neural mechanisms that could mitigate the adverse effects of teleoperation delays. The concept of predictive coding suggests that the brain is not a passive recipient of sensory signals but actively generates predictions about incoming sensory information, updating these predictions as new data arrives (Kilner et al., 2007). This model has profound implications for understanding how simulated feedback might be integrated into neural processes to counteract the disorienting effects of delayed teleoperation. Research by Shadmehr et al. (2010) builds on the predictive coding framework, proposing that the brain's predictive mechanisms allow for smoother motor control by anticipating sensory events. This is particularly relevant when considering the DLPFC and its role in cognitive functions, including the integration of sensory information with motor planning (Abe and Hanakawa, 2009). Simulated feedback, when designed effectively, could harness these predictive mechanisms, potentially reducing the cognitive load and improving motor execution in teleoperation scenarios. The SMA and pre-SMA, regions involved in the initiation and temporal organization of movements (Shima and Tanji, 1998; Zhang et al., 2023), may also benefit from simulated feedback. By providing early sensory cues, simulated feedback could help in “pre-setting” these regions, allowing for more accurate timing predictions and motor responses despite delays (Kilavik et al., 2014). This study mainly relies on fNIRS data for capturing the key neurofunctional characteristics, which will be introduced in the next section.

### 2.2 fNIRS methods in exploring neurodynamic in teleoperation

fNIRS utilizes near-infrared light to monitor brain activity. It operates on the principle that oxygenated and deoxygenated hemoglobin in the brain have distinct absorption spectra in the near-infrared range. When neurons are active, they consume more oxygen, altering the balance between oxygenated and deoxygenated hemoglobin (Zimeo Morais et al., 2018). fNIRS detects these changes, providing an indirect measure of neural activity. This method is advantageous for its non-invasiveness, portability, and relative insensitivity to motion artifacts compared to other neuroimaging techniques, making it suitable for use in diverse settings, including those that simulate real-world teleoperation environments (Tak and Ye, 2014).

Compared to other neuroimaging tools like functional Magnetic Resonance Imaging (fMRI), Electroencephalography (EEG), and Positron Emission Tomography (PET), fNIRS offers unique advantages in the context of teleoperation studies (Abtahi et al., 2020). fMRI, while offering high spatial resolution, is limited by its need for a highly controlled, immobile environment, making it less suitable for dynamic tasks (Ma et al., 2022). EEG, with its excellent temporal resolution, is sensitive to electrical noise and requires complex setups (Parvizi and Kastner, 2018). PET, though powerful in metabolic studies, involves exposure to radioactive tracers, limiting its practicality (Slough et al., 2016). In contrast, fNIRS is more adaptable to naturalistic settings, relatively motion-tolerant, and does not require a strictly controlled environment. This makes fNIRS a more feasible option for teleoperation research compared to these other methods (Balardin et al., 2017). Furthermore, when compared to subjective self-report measures like the NASA Task Load Index (NASA-TLX; Hart and Staveland, 1988), fNIRS provides a more direct, objective measure of brain activity. While questionnaires can capture an operator's self-perceived workload and stress, they are limited by subjective biases and post-task rationalization. fNIRS, on the other hand, allows for the investigation of real-time neural processes underlying task performance (Maior et al., 2014).

fNIRS has proven crucial for uncovering neural functions critical to teleoperation. It effectively measures activity in key areas like the prefrontal cortex, important for executive functions and decision-making, and the primary motor cortex, involved in executing movement commands. These capabilities are essential for understanding how operators manage the complexities and dynamic demands of teleoperation (Sanes and Donoghue, 2000; Euston et al., 2012). Additionally, fNIRS can be used to examine regions associated with sensory integration and processing, such as the prefrontal cortex. This area is crucial in how operators combine visual, auditory, and haptic information during teleoperation. This capability provides deeper insights into the neural mechanisms of multisensory integration, which is essential for managing the complexities of teleoperated environments (Zheng et al., 2023). fNIRS also plays a pivotal role in providing direct insights into the neural mechanisms underpinning operator's motor performance. In the dynamic and often demanding context of teleoperation, where operators must continually adapt to feedback delays and complex control tasks, fNIRS offers a unique observational perspective on cerebral processes. This includes monitoring of changes in cortical blood flow related to cognitive and motor functions, specifically within brain areas like the prefrontal cortex and motor cortex. Such insights are crucial for understanding how operators manage and compensate for sensory feedback delays, enhancing our ability to design more effective teleoperated systems (Zhu et al., 2021). By continuously monitoring brain activity during task performance, fNIRS enables *post-hoc* analysis that allows researchers to observe how variations in teleoperation conditions, such as feedback delays, influence specific brain regions. This analysis is crucial for identifying which aspects of teleoperation are most cognitively demanding and determining how different sensory manipulations can effectively mitigate these challenges.

fNIRS technology is crucial for identifying how different feedback modalities affect operator brain activity. This insight guides the development of user-centered interfaces and training programs, which are tailored to the cognitive demands of teleoperation tasks, enhancing both efficiency and performance.

## 3 fNIRS-based analytical pipeline

### 3.1 fNIRS system

We utilized the NIRx fNIRS device with 16 sources and 15 detectors, plus an additional reference detector at the right pre-auricular point, to filter out extracerebral signals. Operating at a standard 10 Hz sample rate, the device emits near-infrared wavelengths of 760 and 850 nm, enabling differentiation between oxygenated and deoxygenated hemoglobin. Our study concentrated on analyzing data from several key brain regions relevant to robot teleoperation as illustrated in Figure 1.

### 3.2 fNIRS data analysis

**Figure 2:**
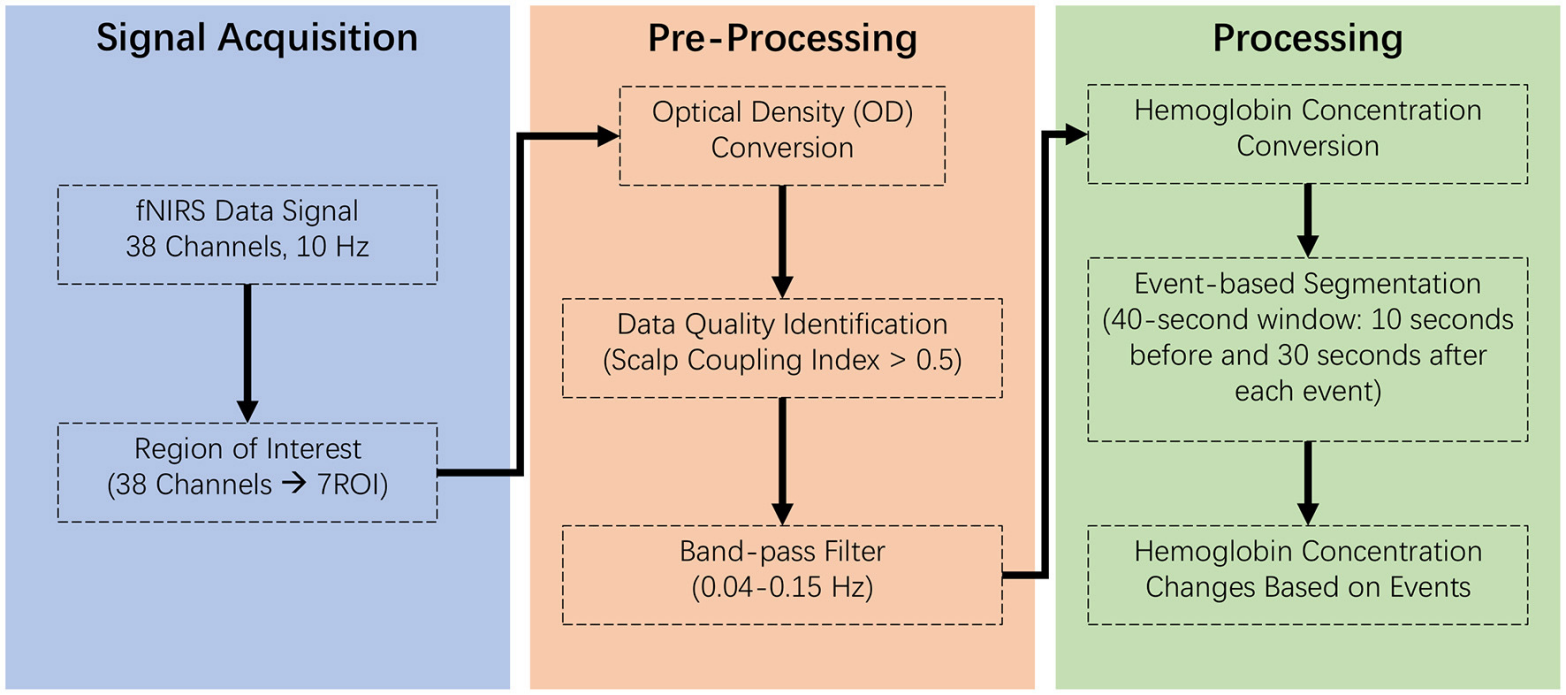
Analytical pipeline for fNIRS data, detailing the sequential steps in the data processing workflow. Signal acquisition: involves collecting raw fNIRS signals under controlled conditions to ensure data integrity and reliability. Preprocessing: entails filtering, correcting, and normalizing the data to remove noise and artifacts, enhancing signal quality for analysis. Processing: consists of applying statistical and computational techniques to extract meaningful patterns and metrics from the preprocessed data, leading to the identification and interpretation of cerebral hemodynamic responses.

We processed raw fNIRS data using MNE-python, effectively removing noise like electronic interference and motion artifacts. This conversion to changes in hemoglobin concentrations (Δ*HbR*) allows us to measure the brain's response to teleoperation delays, assessing cognitive load and decision-making efficiency (Gramfort et al., 2013). The pipeline for fNIRS data analysis is illustrated in Figure 2.

Upon importing the raw fNIRS data, it was converted into optical density (Δ*OD*), a measure reflecting changes in light absorption due to variations in chromophore concentration in the brain tissue (Tak and Ye, 2014). An essential step in ensuring data quality involved the evaluation of the Scalp Coupling Index (SCI), an objective metric quantifying the quality of the optode-scalp connection (Pollonini et al., 2016). The SCI is critical in fNIRS data analysis as it reflects the signal strength and integrity; values < 0.5 typically indicate poor data quality, possibly due to motion artifacts or insufficient contact between the optodes and the scalp. Channels with SCI values below this threshold were excluded from subsequent analysis to maintain the integrity of our dataset.

Following the quality assessment, the optical density data from the fNIRS device underwent a critical filtering process to isolate the neural signals related to cognitive activity from extraneous physiological noise. We employed a finite impulse response (FIR) method, utilizing a bandpass filter within the frequency range of 0.04–0.15 Hz to target various types of noise (Khan et al., 2020; Pinti et al., 2020): *Cardiac Cycles*: Typically, cardiac-related fluctuations occur at frequencies around 1.0–1.5 Hz; *Respiration*: Respiratory patterns generally manifest in the fNIRS signal at frequencies around 0.3 Hz; *Very Low-Frequency Drifts*: Low-frequency drifts in fNIRS data, typically below 0.01 Hz, can arise from slow shifts in sensor positioning or gradual changes in baseline physiological states. The transition band width was set to 0.1 and 0.02 Hz at the high and low cut-off frequencies to ensure a smooth transition between the passband and the stopband, preventing the abrupt cutoff of relevant signals. The high cut-off frequency was designed to exclude high-frequency noise, such as electronic interference or rapid motion artifacts, while the low cut-off frequency was adjusted to remove the slower physiological oscillations without affecting the integrity of the cognitive-related hemodynamic signals.

To measure hemoglobin concentration changes, we utilized the Beer-Lambert Law (Swinehart, 1962). This principle posits that the concentration of a light-absorbing substance within a medium is directly proportional to the length of the light's path through that medium. By applying this law in the context of fNIRS, we estimate changes in oxygenated (HbO) and deoxygenated hemoglobin (Hb) based on the absorption properties of blood, incorporating adjustments for light scattering with a partial pathlength factor. We prioritized HbO as our primary measure due to its enhanced sensitivity to changes in cerebral blood flow, particularly significant in tasks that involve motor execution. This decision is supported by literature indicating HbO's reliable reflection of the brain's response to motor-related demands, as it more directly captures the increase in blood oxygenation following neuronal activation (Obrig and Villringer, 2003; Pereira et al., 2023). These characteristics make HbO a particularly useful indicator in studies focused on motor activities, where accurate measurement of regional brain activation is critical.

In this study, we utilized an event-related analytical approach, focusing on crucial teleoperation tasks such as object pick-up and drop-down. We segmented the fNIRS data into epochs extending from 10 s before to 30 s after each event. This 40-s window was strategically chosen to not only capture the preparatory phase, where participants are actively engaging with the controls to accurately target and maneuver the object, but also to include the post-event period. This approach ensures that we account for significant brain activity initiated by both visual and haptic feedback delays during task execution, which is critical for a comprehensive understanding of cognitive and motor adjustments.

The selected time window also accommodates the inherent delay in hemodynamic responses, commonly referred to as the time-to-peak, which ranges from 2 to 8 s following the stimulus onset (Huppert et al., 2006). Furthermore, the hemodynamic response does not immediately return to baseline after peaking but rather declines gradually over an extended period. This gradual decline can last significantly beyond the peak, necessitating an extended observation window to accurately capture the entire hemodynamic curve (Lindquist et al., 2009; Amiri et al., 2014; Duarte et al., 2023). Baseline levels were established during a separate 5-min measurement phase prior to task engagement, ensuring that the fNIRS data collected during tasks are accurately reflective of changes due to task-specific brain activity.

To maintain data integrity, a thorough cleaning process was implemented to remove physiological interferences, such as those caused by heartbeats and respiration (Pinti et al., 2020). This meticulous approach to data preparation ensures that our analysis remains focused on the brain activity directly linked to each task performance. Averaging data across all phases of the experiment could potentially obscure these detailed event-specific hemodynamic patterns, particularly given the longer periods of lower neural activity that occur between the task events.

For our primary measure, we calculated the mean change in oxygenated hemoglobin (delta HbO) within this 40-s window for each event, thus providing a detailed view of the brain's response to each specific task action. This approach was chosen to capture the hemodynamic responses associated with the specific tasks or stimuli in our experiment, providing a direct measure of the cerebral blood flow changes over time. This averaging is intended to stabilize the signal against short-term fluctuations and highlight more sustained changes in brain activity that are directly relevant to task performance.

While this method effectively captures the overall hemodynamic pattern related to specific events, we acknowledge that it averages out finer temporal details within these windows. Some of the finer temporal dynamics, particularly those within shorter time intervals, are not distinctly represented. Future studies might benefit from incorporating more granular time-series analyses, such as General Linear Model (GLM) approaches, which could provide additional insights into the precise timing and magnitude of neural responses. Such analyses would complement our current findings by offering a detailed temporal resolution of neural activity patterns, enhancing our understanding of the neural underpinnings in teleoperation tasks.

## 4 Materials and methods

### 4.1 Overview

The study was approved by the Institutional Review Board (IRB) of the University of Florida, Gainesville, FL, USA (No. IRB202100257). Written informed consents were obtained from all participants in full accordance with the ethical principles of the relevant IRB guidelines and regulations. All methods were carried out in accordance with relevant guidelines and regulations. The following inclusion criteria were applied: (1) age ≥ 18 years; (2) no known physical or mental disabilities; (3) no known musculoskeletal disorders.

### 4.2 Experiment design

The main task in the human-subject experiment was an object manipulation task. The experiment was designed as a within-participant experiment, i.e., each participating subject experienced four conditions. To avoid learning effects, the sequence order was shuffled for each subject. The performance data (time and accuracy), motion data (moving trajectory), eye tracking data (gaze focus and pupillary size), and neurofunctional data (measured by fNIRS) were collected. Participating subjects were also requested to report their perceived delays, to compare them with the actual delays. Before the experiment, each participant was required to fill out a form of demographic survey, and the consent form approved by UF's IRB office. Then they would take a training session, to familiarize themselves with the use of VR. Afterwards, participants were required to take a break by sitting quietly with all sensors on. This break session was for collecting baseline data (e.g., pupillary diameter and fNIRS baseline), and to remove possible impacts of the training session. After each experiment trail, participants were promoted to fill out questionnaires related to NASA TLX and trust.

**Figure 3:**
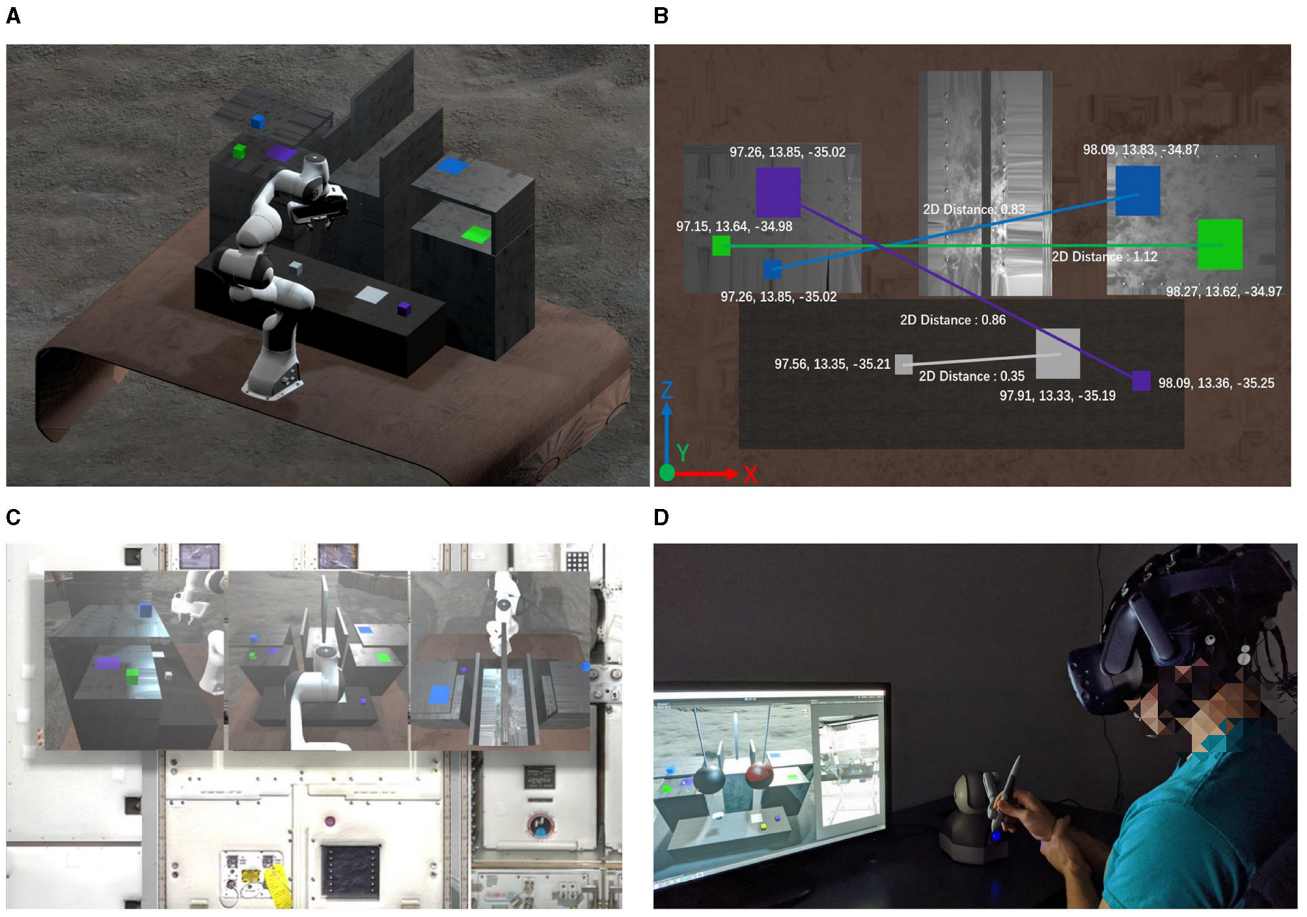
The layout of the object manipulation task in human-subject experiments. **(A)** 3D perspective of the experimental scene; **(B)** Objects and targets setup; **(C)** First person view of the participants; **(D)** Participants completed the experiment using haptic feedback device.

Participants needed to interact with four colored cubes: gray, green, blue, and purple. Each cube aligned with a target with the same color, requiring participants to accurately move these cubes following a predefined sequence: gray, green, blue, and then purple. The sequenced tasks were systematically structured to gradually increase in complexity and challenge. In this setting, each cube's path to its corresponding target was blocked by various obstacles, which carefully integrated into the task environment. These obstacles vary in size and position, adding to the complexity of the task and representing different locomotor challenges that participants had to contend with as illustrated in Figure 3. In total, each participant needs to complete 10 trails (as illustrated in Table 1) and each trail has four blocks needed to move.

**Table 1:** Feedback delays correspond to each condition.

**Condition**	**Visual delay (sec)**	**Haptic delay (sec)**
Standard	0	0
Anchoring	0.25	0
Anchoring	0.5	0
Anchoring	0.75	0
Synchronous	0.25	0.25
Synchronous	0.5	0.5
Synchronous	0.75	0.75
Asynchronous	0.5	0.25
Asynchronous	0.75	0.25
Asynchronous	1.0	0.25

To minimize the effects of fatigue, our experimental session was structured to be ~1 h per participant, including all preparation and breaks. Device Setup and Training (10 min): Participants spent around 10 min wearing the fNIRS device and getting familiarized to the virtual reality (VR) environment to ensure comfort and reduce anxiety or fatigue during the experiment. Baseline Measurement (5 min): A 5-min break was provided to establish a fNIRS baseline, allowing participants to rest before engaging in the tasks. Task Conditions (average 3 min each): Each experimental condition was designed to last an average of 3 min. These short, manageable intervals helped maintain participant focus and energy. Rest Periods: Regular 1-min breaks were incorporated between conditions to provide participants with time to relax and minimize fatigue.

Additionally, to address potential order effects and ensure that fatigue did not disproportionately affect any single condition, we employed a counterbalancing approach by shuffling the sequence of conditions for each participant. This approach was aimed at distributing any potential fatigue effects evenly across all conditions and ensuring that no specific task was consistently encountered at the end of the session.

When dissecting the delay, it is categorized into haptic feedback delay (Δ_*haptic*_), and visual feedback delay (Δ_*visual*_). As illustrated in Table 1, our experiment was based on four sensory manipulation conditions as follows:

*Condition 1: Standard condition*: Δ_*haptic*_ = Δ_*visual*_, resulting in instantaneous haptic and visual feedback. In this real-time interaction scenario, the operator receives immediate multisensory feedback post-action initiation.

*Condition 2: Anchoring*. Δ_*haptic*_ = 0 while Δ_*visual*_ changes. Due to the intrinsic delays in visual feedback, real-time haptic responses are generated post-action initiation based on the simulated force feedback (e.g., inertia, resistance, and vibration) at the local workstation.

*Condition 3: Synchronous*. Both Δ_*haptic*_ and Δ_*visual*_ are intentionally subjected to a synchronized delay in order to promote multisensory alignment and enhance the coherence of perceptual experiences through the alignment of sensory modalities.

*Condition 4: Asynchronous*. This condition embodies variable delays in sensory feedbacks, presenting a realistic and challenging scenario in which perceptible delays influence the initiation and reception of haptic and visual feedbacks.

The standard condition is intended to serve as the ground truth or baseline for comparison with other experimental conditions. Specifically, this condition is conducted under optimal conditions where there are no visual or haptic delays, providing an unaltered scenario that represents the ideal state of teleoperation performance.

### 4.3 Experiment platform

Building upon our detailed system design presented in Du et al. (2023), this section offers a concise overview of the key components of our teleoperation system, focusing on the VR system, its integration with various elements, and the implementation of delay coding functions.

Central to our teleoperation system is an advanced Virtual Reality (VR) setup, providing a fully immersive simulation environment developed in Unity. This platform replicates the physical dynamics and robot interactions with high fidelity, ensuring a realistic teleoperation experience. Another critical element in our system is the seamless integration between the Robot Operating System (ROS) and the Unity-based VR environment, facilitated by ROS#. This connection allows for real-time synchronization between the virtual environment and the physical robot, ensuring that any action taken in the VR space is instantly mirrored in the robot's movements.

To enhance the realism and interactivity of the VR environment, we incorporated the Touch X haptic controller. This device provides haptic feedback, replicating the physical sensations of manipulating objects or encountering resistance, crucial for tasks requiring fine motor control. The haptic feedback system is intricately coded to respond to both the operator's actions and the simulated physics of the VR environment, creating a cohesive and immersive experience. Finally, recognizing the impact of feedback delays on teleoperation, our system architecture includes specially developed coding functions to simulate various delay scenarios. Both visual and haptic feedback can be intentionally delayed, allowing us to study the operator's adaptability and performance under different sensory delay conditions.

While our VR task provides valuable insights into the neurofunctional and motor control challenges in teleoperation, it is distinct from surgical teleoperation, which involves additional complexities such as biological variability and higher stakes in terms of patient safety. Our findings contribute to a broader understanding of teleoperation in non-medical contexts, offering implications for the design and training of teleoperated systems in industrial and rescue operations. Future research could explore how these insights might translate to the more nuanced requirements of surgical environments.

### 4.4 Data collection methods

Optimal data collection quality for fNIRS requires careful preparation. Participants were advised to ensure their hair was clean and free from products that could obstruct the fNIRS sensors, and to avoid hairstyles or accessories that might disrupt the cap's placement. This preparation stage was critical for enhancing sensor-skin contact and the fidelity of the collected data, enabling a more accurate assessment of the cortical activity associated with the cognitive demands of the task. The stability of the experimental conditions, including controlled lighting and the participant's stationary posture while operating the haptic device, ensured that data integrity was maintained.

In the beginning, participants were asked to sign an informed consent form and fill out a background questionnaire about their age, gender, and VR experience. The experimental scene and content of each phase were the same. The sequence of tasks under different conditions was shuffled to eliminate the learning effects. The training session was designed to familiarize participants with the VR system and interactions within the virtual environment. Each participant was instructed to be acquainted with the devices (VR headset and haptic controller) and the virtual environment. Then, participants were given instructions about how to use the haptic controller to pick up and place the objects. After the training session, participants were asked to perform the pick-up and place task based on the virtual pipe skid system. After each phase, participants provided feedback through NASA TLX questionnaires.

During the experiment, participants were required to precisely control the robot gripper to stably grasp the cubes without knocking them away. Once successfully grasped, they should control the robot gripper past the obstacles and accurately place them on the corresponding target plate. The accuracy of the cube's positioning on the target is crucial, as it is a key metric for evaluating participants' operational performance. The use of visual and haptic feedback delays in the experimental design was critical for simulating the temporal challenges inherent in teleoperation tasks. These delays required the participants to rely on their cognitive adaptability, a phenomenon that conventional behavioral metric might not fully capture. Employing fNIRS allowed us to measure the operator's brain activities in response to sensory feedback delays, providing objective data on the neural correlates of delay adaptation in teleoperation. This technique helped us to elucidate the fundamental neurological mechanisms impacted by delays and the cognitive strategies employed by operators during task performance.

To ensure robust statistical analysis, we initially assessed the distribution of each variable for normality. Variables not normally distributed were transformed using a logarithmic transformation to achieve normality. We then verified that normally distributed variables had homogeneous variances. For variables meeting these assumptions, we employed repeated measures Analysis of Variance (ANOVA) to analyze parametric study measures. For data that did not meet parametric assumptions, we conducted Signed Rank Wilcoxon tests to identify significant differences.

Prior to the main study, we conducted a pilot with five participants to refine our experimental procedures and perform an initial power analysis. This analysis, conducted using the open-source library Pingouin (Vallat, 2018), was based on Vallat's recommendations, considering the condition as the between-group factor and placement error and time on task as dependent variables. The power analysis indicated effect sizes of 0.50 for placement error and 0.31 for time on task, determining minimum sample sizes of 4 and 8, respectively, to achieve a power of 0.8 with a Type I error probability of 0.05. To enhance the reliability of our findings, we expanded our sample to 41 healthy subjects. A subsequent power analysis incorporating all participants demonstrated a statistical power of 0.997, indicating a very high likelihood of detecting significant differences among the conditions in our ANOVA tests. This robust sample size greatly increases our confidence in the statistical validity and reliability of our results.

## 5 Results

### 5.1 Participants

We recruited a total of 41 subjects for this experiment. The demographic information includes the gender, age group, major, and VR experience of participants are illustrated in Table 2. All participants reported that they were right-handed and did not have any known motor disorders or a history of neurological abnormalities.

**Table 2:** Demographic information of the participants.

		**Number**	**Percentage**
Gender	Male	26	63.41%
	Female	15	36.59%
Age group	18–24	14	34.15%
	25–30	24	58.53%
	31 and older	3	7.31%
Major	Engineering (civil, coastal, construction, mechanical, and related)	18	43.90%
	Non-engineering	23	56.10%
VR experience	Experience with VR	12	29.27%
	Non-experience with VR	29	70.73%

### 5.2 Performance results

**Figure 4:**
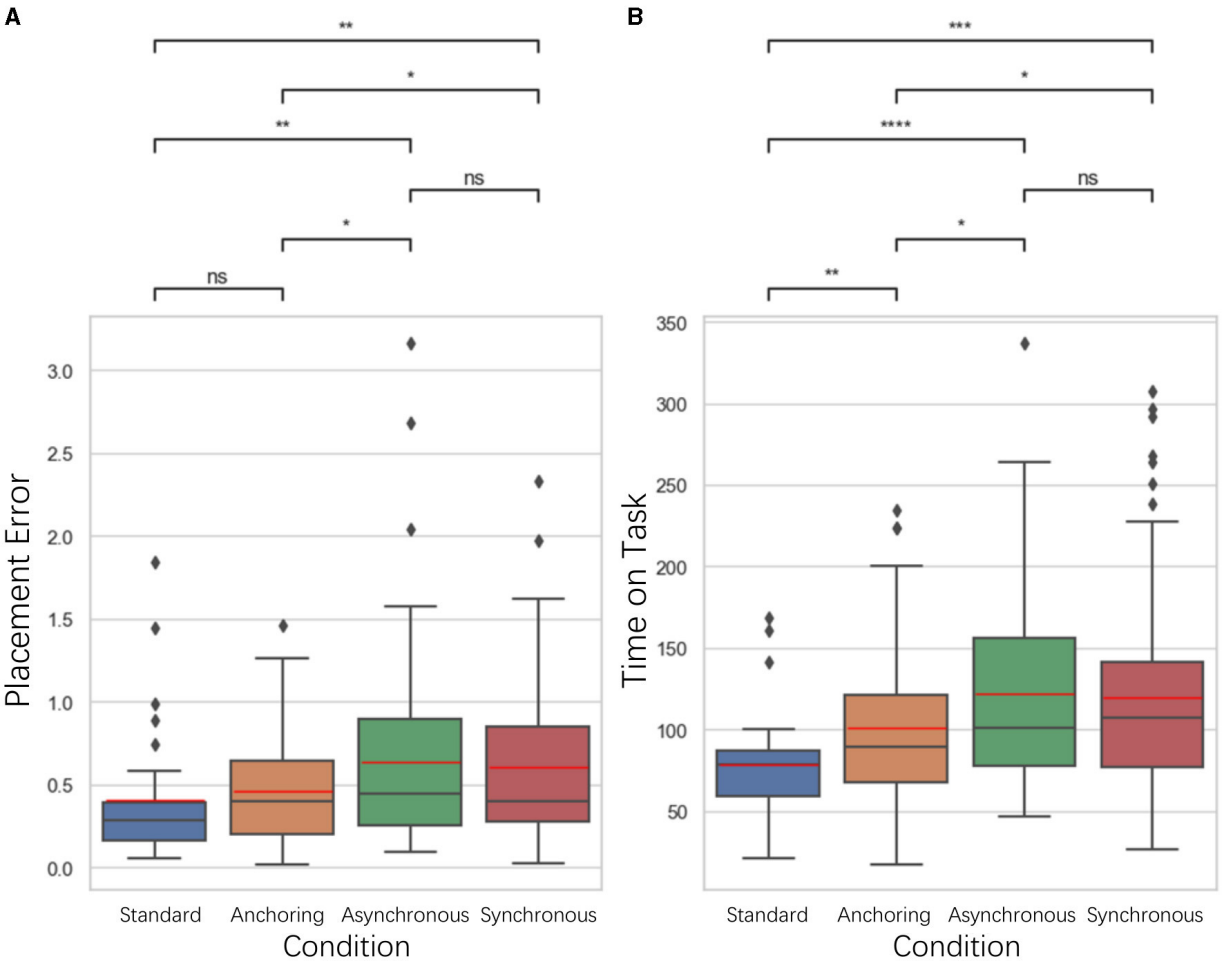
Statistical analysis results of **(A)** placement error and **(B)** time on task comparison. ^*^Indicates statistically significant change (n.s., no significant difference, ^*^p < 0.05, ^**^p < 0.01, ^***^p < 0.001, ^****^p < 0.0001).

**Figure 5:**
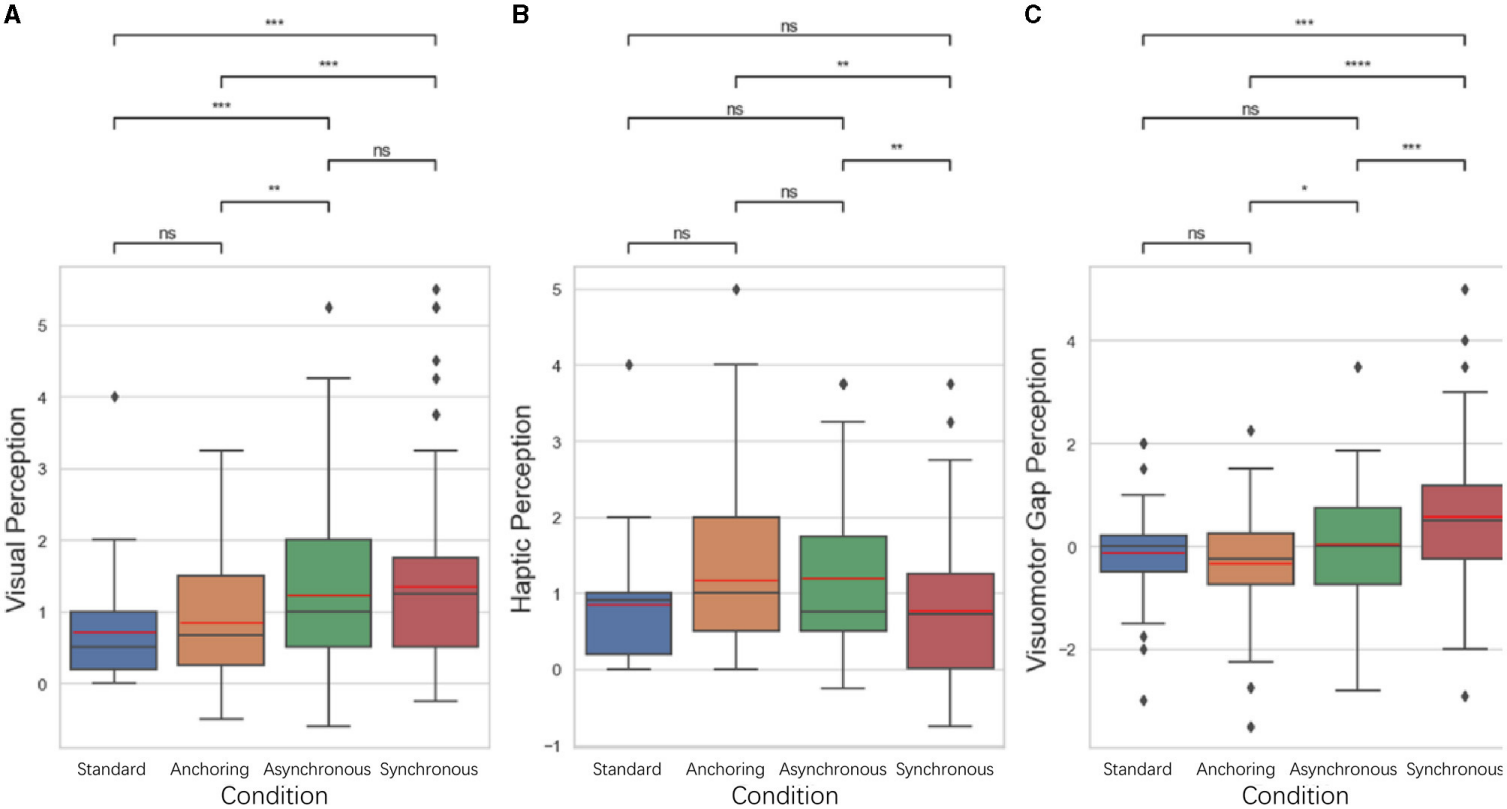
Statistical analysis results of perception performance. **(A)** Visual perception difference; **(B)** haptic perception difference; **(C)** visuomotor gap perception difference. ^*^Indicates statistically significant change (n.s., no significant difference, ^*^p < 0.05, ^**^p < 0.01, ^***^p < 0.001, ^****^p < 0.0001).

**Figure 6:**
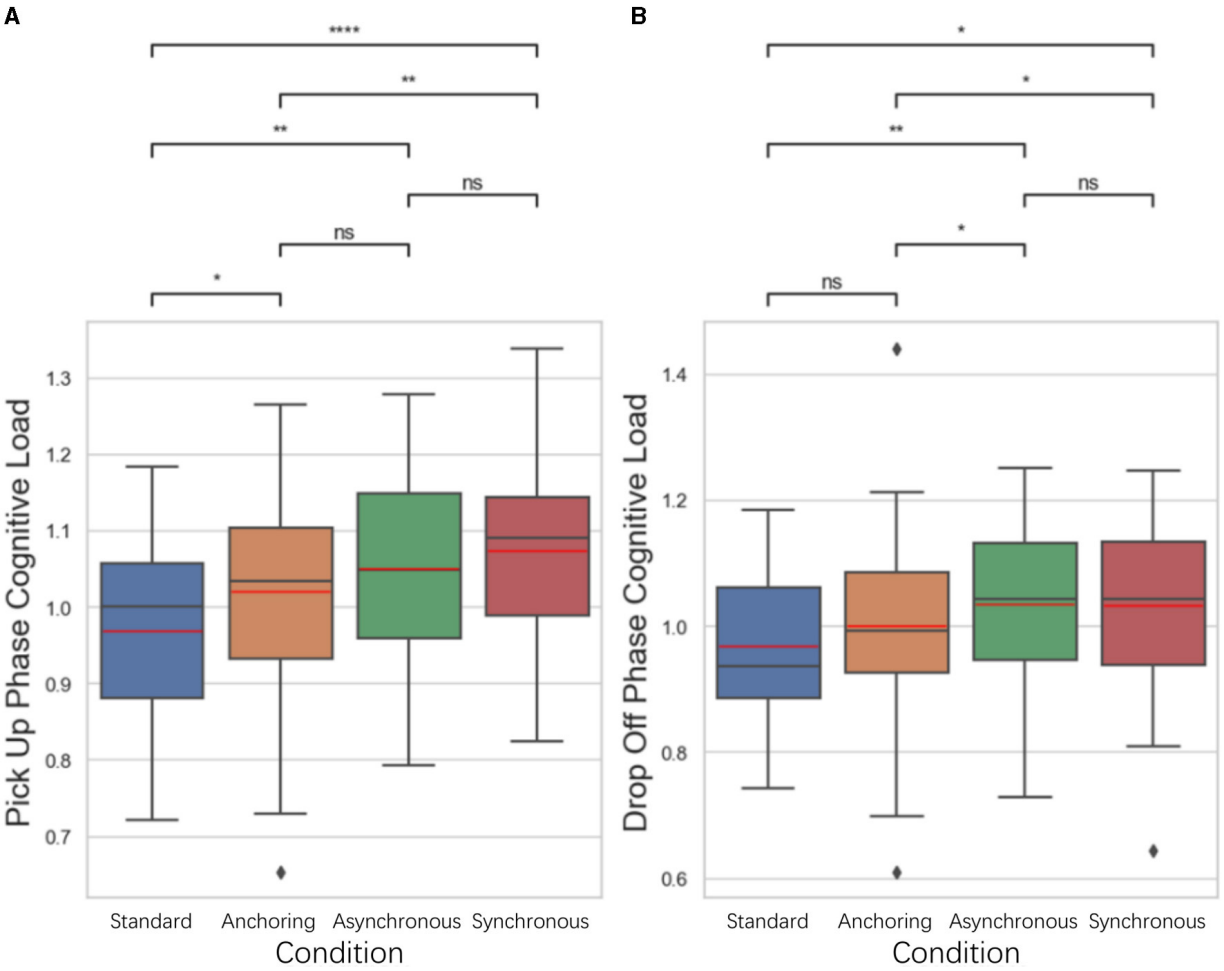
Statistical analysis results of cognitive load changes in **(A)** object pickup and **(B)** drop-off stages. ^*^Indicates statistically significant change (n.s., no significant difference, ^*^p < 0.05, ^**^p < 0.01, ^***^p < 0.001, ^****^p < 0.0001).

**Figure 7:**
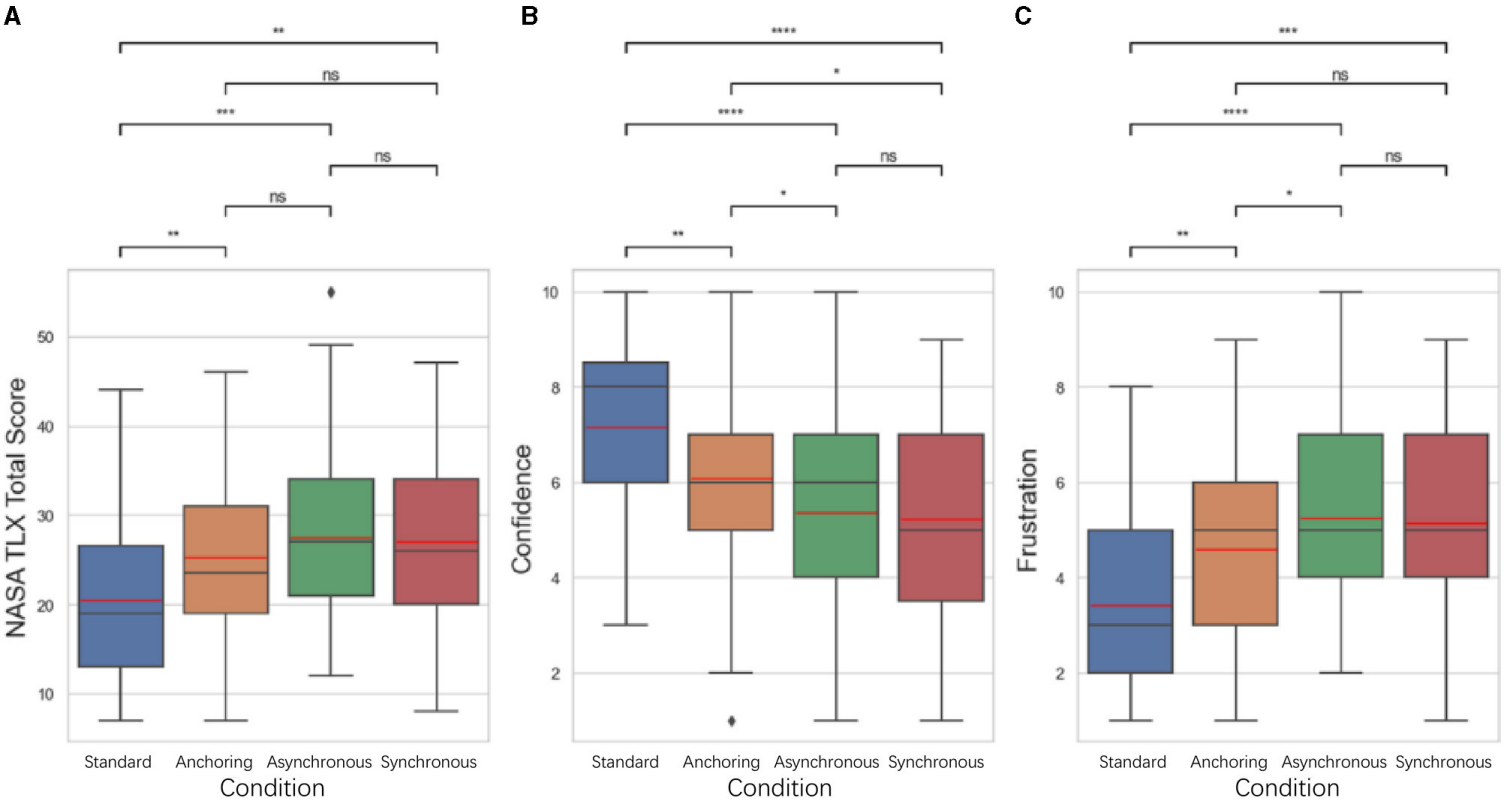
Statistical analysis results of NASA TLX related to **(A)** total score (when calculating the total score, 10-*Confidence score* is used as the calculation parameter), **(B)** self-confidence level, and **(C)** frustration level for delays up to 1 s. Other NASA TLX results are not shown because of the insignificant difference among the conditions. ^*^Indicates statistically significant change (n.s., no significant difference, ^*^p < 0.05, ^**^p < 0.01, ^***^p < 0.001, ^****^p < 0.0001).

In our previous study Du et al. (2023), we investigated various performance metrics to determine the influence of delayed feedback in teleoperation. The placement error, time on task, and cognitive load during pick-up and drop-off phases were evaluated using pupil size as a physiological indicator. Subjective assessments were also employed through the NASA-TLX questionnaire to measure the perceived workload and stress levels of participants. Figures 4–**7**, **10**–**13** is the comparison analysis results among four conditions: Standard (Δ_*haptic*_ = Δ_*visual*_), Anchoring (Δ_*haptic*_ = 0 while Δ_*visual*_ changes), Synchronous (Both Δ_*haptic*_ and Δ_*visual*_ are intentionally subjected to a synchronized delay), and Asynchronous (variable delays in sensory feedbacks). ^*^Indicates statistically significant change (n.s., no significant difference, ^*^p < 0.05, ^**^p < 0.01, ^***^p < 0.001, ^****^p < 0.0001). The boxplot shows the distribution of the data being analyzed. The bottom and top of the box represent the 25 and 75th percentiles, respectively, while the line inside the box denotes the median (50th percentile). The whiskers extend to the 1.5 IQR (interquartile range), and the error bars indicate the 95% confidence intervals (CI = 95%), providing a statistical measure of the precision of the sample mean. Outliers are represented as solid circles. The black horizontal line in the box plots below represents the median of the data and the red line represents the mean of the data.

For placement error, we measured it as the Euclidean distance between the actual placement of the cube and the center of the target location. As illustrated in Figure 4, the results indicate that for the placement error, the standard condition is significantly better than asynchronous condition (*p* = 0.007) as well as synchronous condition (*p* = 0.004); the anchoring condition is significantly better than the asynchronous condition (*p* = 0.043) and the synchronous condition (*p* = 0.032). There is no significant difference between the standard and anchoring (*p* = 0.168), the asynchronous and synchronous condition (*p* = 0.892). Time on Task is the difference between the end time and the start time of the task. The results also indicate significant differences between the standard and anchoring condition (*p* = 0.009), asynchronous condition (*p* < 0.001), synchronous condition (*p* < 0.001); and between anchoring and asynchronous condition (*p* = 0.018) as well as the synchronous condition (*p* = 0.049). There is no significant difference between the asynchronous and synchronous condition (*p* = 0.741).

About time perception, we focused on examining three time perception metrics: visual perception difference, haptic perception difference, and visuomotor gap perception difference.

**Perceived visual delay:** This is the delay that participants perceive between initiating an action and seeing the result visually. It is measured by asking participants to estimate the visual delay they experience during each phase of the experiment.

**Actual visual delay:** This is the delay objectively introduced in the visual feedback within the teleoperation system. It is a controlled variable set by the experiment to simulate different conditions of teleoperation latency.

**Perceived haptic delay:** Similar to perceived visual delay, this is the delay that participants report feeling between initiating an action and receiving haptic feedback. This is measured through participant self-report after each experimental phase.

**Actual haptic delay:** This is the objectively measured delay between the initiation of an action and when the haptic feedback is provided by the system. Like the actual visual delay, this is a predefined variable controlled throughout the experiment to simulate various feedback scenarios.

**Perceived visuomotor gap:** This refers to the gap that participants perceive between the visual and haptic delays. It is calculated as the difference between perceived visual delay and perceived haptic delay.

**Actual visuomotor gap:** This is the actual difference between the visual and haptic delays as programmed into the teleoperation system. It is calculated as the difference between the actual visual delay and the actual haptic delay.

Visual perception difference (Equation 1) is defined by the difference between the perceived visual delay (*Delay*_*vp*_) and the actual visual delay (*Delay*_*va*_) in a phase, i.e.,


(1) $$\begin{array}{l} \Delta_{v}=Delay_{vp}-Delay_{va} \end{array}$$

Haptic perception difference (Equation 2) is defined by the difference between the perceived haptic delay (*Delay*_*hp*_) and the actual haptic delay (*Delay*_*ha*_) in a phase, i.e.,


(2) $$\begin{array}{l} \Delta_{h}=Delay_{hp}-Delay_{ha} \end{array}$$

Note there were cases when there was a gap between the visual delay and the haptic delay, which we call visuomotor gap. We are also interested in the perception of the visuomotor gaps in different conditions. Visuomotor perception difference (Equation 3) is defined by the difference between the perceived visuomotor gap (*Gap*_*p*_) and the actual visuomotor gap (*Gap*_*a*_) in a phase, i.e.,


(3) $$\begin{array}{l} \Delta_{gap}=Gap_{p}-Gap_{a} \end{array}$$

The results show that for visual perception difference, the standard is significantly lower than asynchronous condition (*p* < 0.001) and synchronous condition (*p* < 0.001); anchoring condition is significantly lower than the asynchronous condition (*p* = 0.003) as well as the synchronous condition (*p* < 0.001). There is no significant difference between the standard and anchoring (*p* = 0.448), the asynchronous and synchronous condition (*p* = 0.506). For the haptic perception difference, the results indicate that synchronous condition is significantly lower than anchoring condition (*p* = 0.003) as well as asynchronous condition (*p* = 0.001). There is no significant difference between the standard and anchoring condition (*p* = 0.091), asynchronous condition (*p* = 0.090), synchronous condition (*p* = 0.052); between anchoring and asynchronous condition (*p* = 0.098). For the visuomotor gap perception difference, synchronous condition is significantly larger than standard condition (*p* < 0.001), anchoring condition (*p* < 0.001), asynchronous condition (*p* < 0.001); anchoring condition is lower than asynchronous condition (*p* = 0.024). There is no significant difference between standard and anchoring condition (*p* = 0.237) and asynchronous condition (*p* = 0.534). In the synchronous condition, where both visual and haptic feedbacks were delayed identically, we observed a surprisingly large visuomotor perception gap. This could be attributed to several interrelated factors:

Integration and expectation of sensory inputs: Participants in synchronous conditions might process aligned sensory delays with heightened sensitivity, leading to an exaggerated perception of discrepancies. This sensitivity is potentially compounded by precise expectations of temporal alignment, where any minor deviation in synchronization between seen and felt stimuli could be perceived as a significantly larger gap.

Lack of adaptive calibration: Unlike asynchronous conditions where participants might gradually adapt to staggered sensory delays, the synchronous setting does not encourage such adaptive strategies. Without the need to adjust to differing times of sensory inputs, the brain may not calibrate as effectively to the delays, maintaining a consistent perception of a larger gap. This lack of adaptation could result in a more pronounced discrepancy between expected and actual sensory feedback, accentuating the perceived visuomotor gap.

These insights into the cognitive processing of synchronized sensory feedback highlight the complexity of human perception under controlled delay conditions. Further research is warranted to delineate the specific neural and cognitive mechanisms that contribute to these perceptions, potentially using more nuanced psychophysical tests or neuroimaging to track how the brain integrates and responds to synchronous vs. asynchronous stimuli.

For cognitive load, we developed a novel approach to evaluate participants' real-time cognitive load based on their pupillary diameter data (mm) collected by eye trackers. We divided the data of each trail into object pick-up stage and object drop-off stage. As illustrated in Figure 6, for the pick-up stage, the standard condition has lower cognitive load than anchoring condition (*p* = 0.032), asynchronous condition (*p* = 0.003), synchronous condition (*p* < 0.001); anchoring condition have lower cognitive load than synchronous condition (*p* = 0.004). There is no significant difference between anchoring and asynchronous condition (*p* = 0.086), between asynchronous and asynchronous condition (*p* = 0.276). For the drop-off stage, the results indicate that the standard also better than asynchronous condition (*p* = 0.006) and synchronous condition (*p* = 0.012); the anchoring condition has lower cognitive load than asynchronous condition (*p* = 0.048) and the synchronous condition (*p* = 0.045). There is no significant difference between the standard and anchoring condition (*p* = 0.178), between the asynchronous and synchronous condition (*p* = 0.983).

The NASA-TLX results shown in Figure 7. The results indicate that for total score, standard condition have the lowest cognitive load compared to anchoring condition (*p* = 0.021), asynchronous condition (*p* = 0.006), synchronous condition (*p* = 0.024); There is no significant difference between anchoring and asynchronous condition (*p* = 0.470) as well as the synchronous condition (*p* = 0.843); between the asynchronous and synchronous condition (*p* = 0.632). For confidence level, standard condition also shows highest confidence level compared to anchoring condition (*p* = 0.007), asynchronous condition (*p* < 0.001), synchronous condition (*p* < 0.001); anchoring condition is significantly higher than asynchronous condition (*p* = 0.024) as well as the synchronous condition (*p* = 0.019). There is no significant difference between the asynchronous and synchronous condition (*p* = 0.829). For frustration level, standard condition still better than anchoring condition (*p* = 0.004), asynchronous condition (*p* < 0.001), synchronous condition (*p* < 0.001); anchoring shows lower frustration level than asynchronous condition (*p* = 0.033). There is no significant difference between anchoring and synchronous condition (*p* = 0.110), between asynchronous and synchronous condition (*p* = 0.694).

Participants in the standard condition reported higher levels of self-confidence and lower levels of frustration compared to other conditions, with anchoring also outperformed the synchronous and asynchronous conditions. The results from these metrics provided an initial understanding of the operational performance and cognitive states of operators under different feedback conditions.

Building upon this foundation, the present study delves deeper into the cognitive activities in different brain areas. By using fNIRS, we aim to demonstrate the specific brain regions engaged during teleoperation tasks, thereby providing a more refined perspective on the neural correlates of performance and brain activation. This approach allows us to pinpoint the hemodynamic responses in areas critical for decision-making, sensorimotor coordination, and time perception, factors that are critical to managing the challenges posed by feedback delays in teleoperation.

### 5.3 fNIRS results

**Figure 8:**
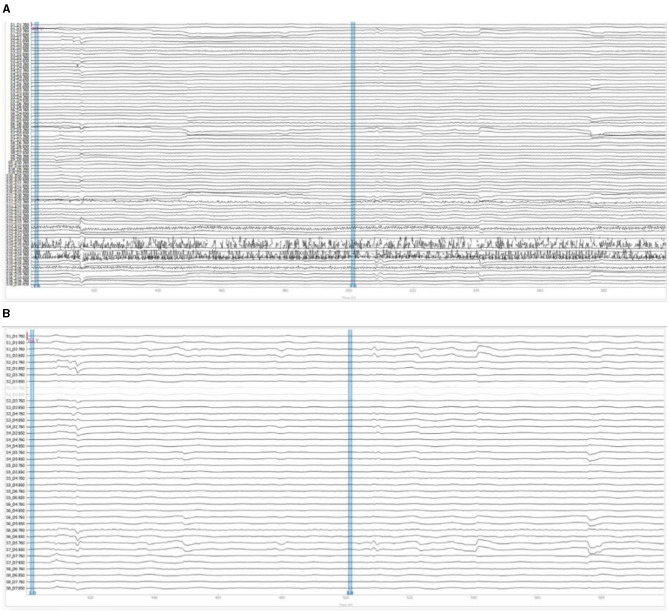
fNIRS signal preprocessing example of participant #11: **(A)** raw optical density signals; **(B)** filtered optical density signals.

Figure 8A illustrated the raw OD data as initially recorded during the teleoperation tasks and Figure 8B illustrated the filtered OD data. The SCI was used to identify and exclude channels with insufficient signal quality, which show as lighter lines in filtered data. The remaining channels were then subjected to a bandpass filter, carefully designed to remove physiological noise such as cardiac and respiratory influences while preserving the signals pertinent to cognitive activity. These filtered OD values were then further processed to derive the concentration changes of oxyhemoglobin based on Beer-Lambert Law.

**Figure 9:**
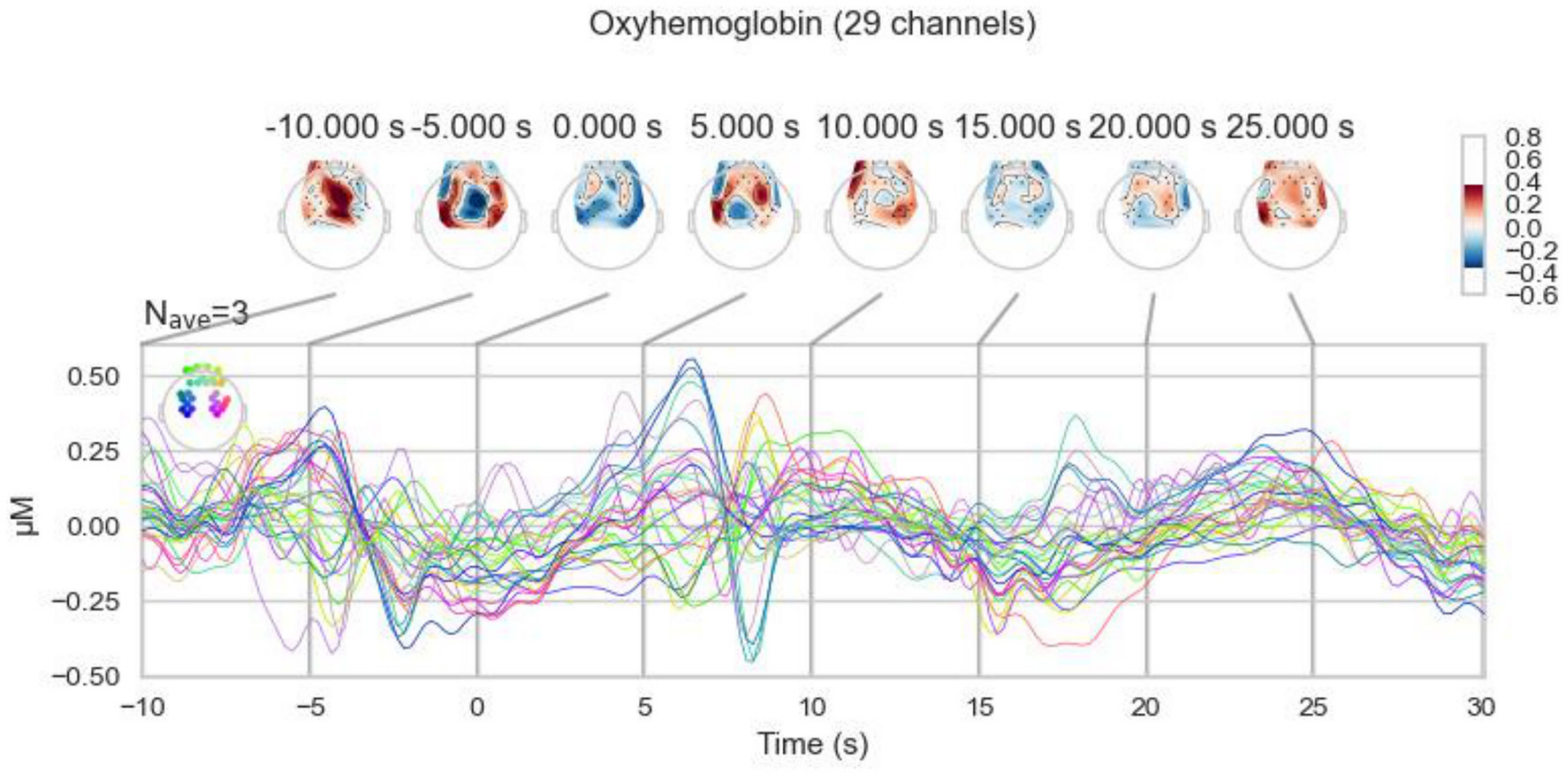
Oxyhemoglobin concentration changes of the pick-up event for participant #11.

To analyze the brain activities to task events in teleoperation, we segmented the processed fNIRS data into specific epochs. Each epoch ranges from 10 s before to 30 s after the events of object pick-up and drop-off. Figure 9 presents an example of this segmentation, showcasing data from participant #11 during a pick-up event. The figure visualizes the changes in oxyhemoglobin concentration, reflecting the brain's hemodynamic response during this critical phase of the task.

To comprehensively evaluate the impact of different teleoperation conditions on brain activity, we conducted a statistical analysis of the oxyhemoglobin concentration across various brain areas. We employed the Kruskal-Wallis test, a non-parametric method used to determine if there are statistically significant differences between the groups. It is especially useful when our data does not follow a normal distribution, which is often the case in real-world data. The test essentially assesses whether one group is stochastically larger than the other and provides a *p*-value that we can use to test our hypothesis.

#### 5.3.1 Anterior prefrontal cortex results

**Figure 10:**
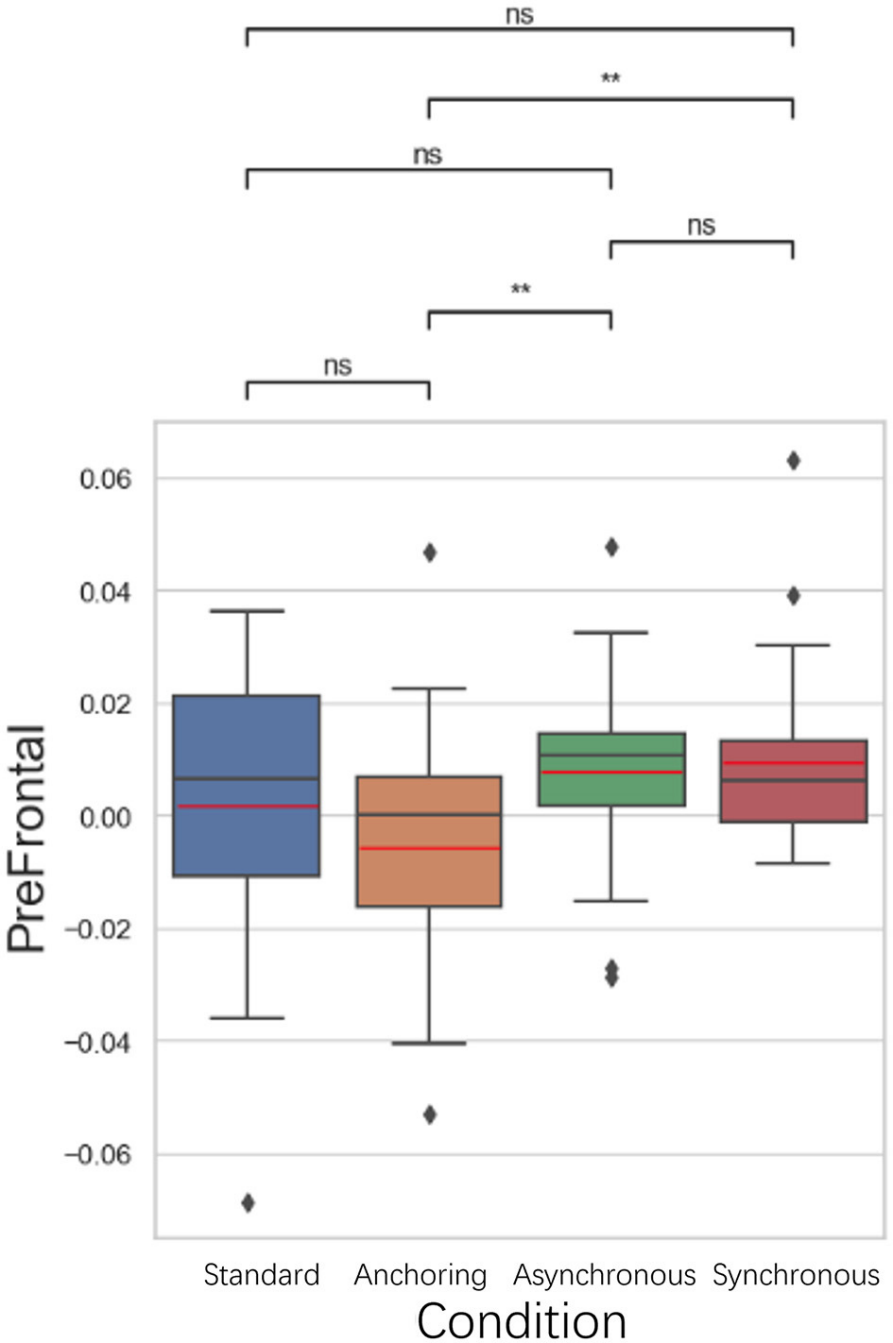
Statistical analysis results of oxyhemoglobin concentrations changes in anterior prefrontal cortex. ^*^Indicates statistically significant change (n.s., no significant difference, ^*^p < 0.05, ^**^p < 0.01, ^***^p < 0.001, ^****^p < 0.0001).

As illustrated in Figure 10, in the anterior prefrontal cortex, known for its role in executive functions and decision-making, the anchoring condition showed lower brain activation compared to the asynchronous (*p* = 0.005) and synchronous conditions (*p* = 0.006). There is no significant difference between standard and anchoring conditions (*p* = 0.126), asynchronous conditions (*p* = 0.062), synchronous conditions (*p* = 0.063); between asynchronous and synchronous conditions (*p* = 0.883). This could suggest that immediate haptic feedback, even when visual feedback is delayed, may help reduce the cognitive demands associated with integrating sensory information and making decisions. This reduction in brain activation could facilitate more efficient task performance, as the operator may rely more on the sense of touch, which is less affected by the delays.

#### 5.3.2 Dorsolateral prefrontal cortex results

**Figure 11:**
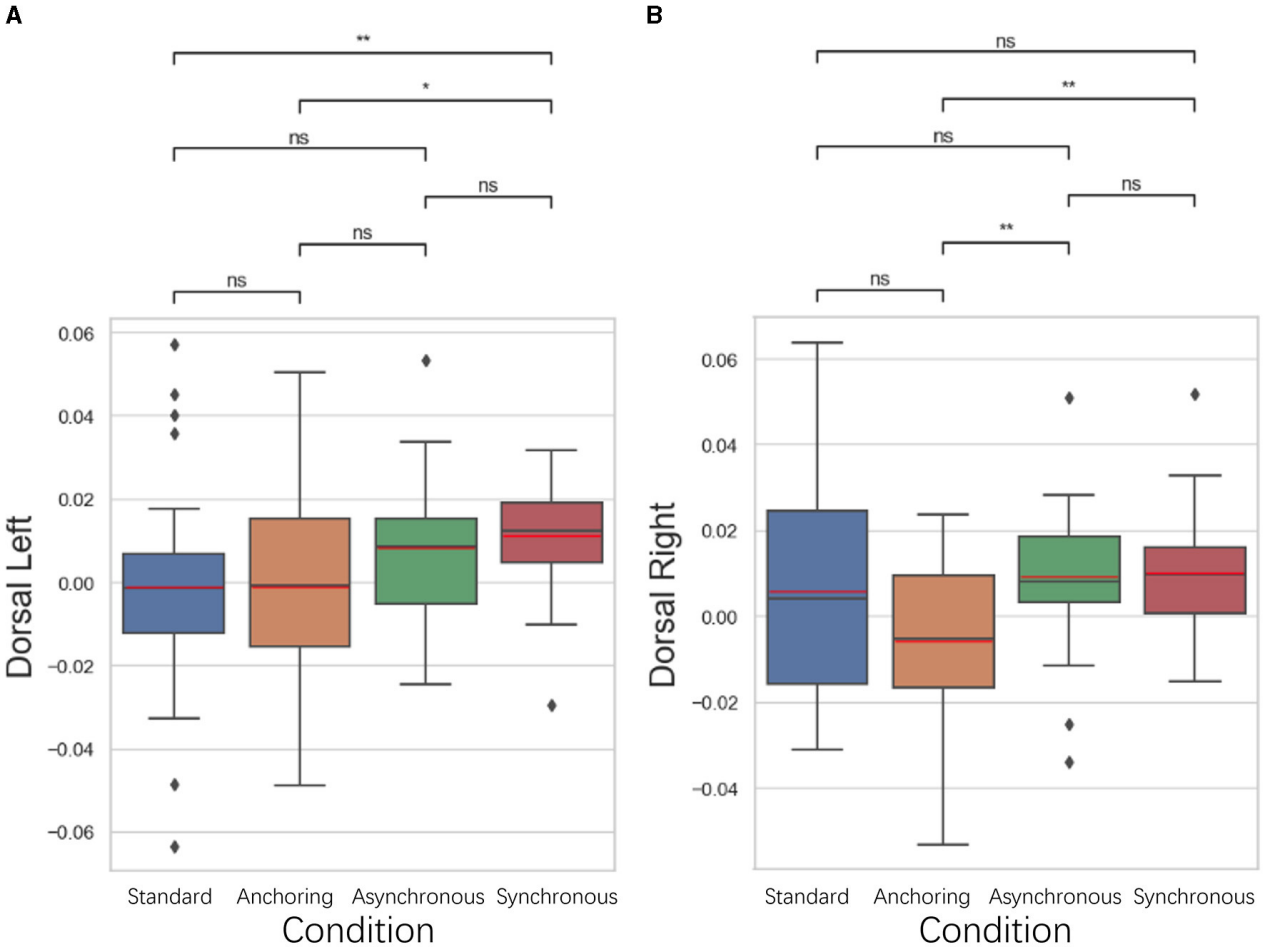
Statistical analysis results of oxyhemoglobin concentrations changes in **(A)** left dorsolateral prefrontal cortex and **(B)** right dorsolateral prefrontal cortex. ^*^Indicates statistically significant change (n.s., no significant difference, ^*^p < 0.05, ^**^p < 0.01, ^***^p < 0.001, ^****^p < 0.0001).

As illustrated in Figure 11, in the dorsolateral prefrontal cortex, associated with motor planning, working memory, and the cognitive aspects of time perception, exhibited a pattern of reduced brain activation in the anchoring condition. The left dorsolateral prefrontal cortex displayed a lower brain activation in both the standard (*p* = 0.006) and anchoring (*p* = 0.017) conditions than in the synchronous condition. There is no significant difference between standard and anchoring conditions (*p* = 0.993) as well as asynchronous conditions (*p* = 0.073); between anchoring and asynchronous conditions (*p* = 0.095); and between asynchronous and synchronous conditions (*p* = 0.392). The right dorsolateral prefrontal cortex exhibited a lower brain activation in the anchoring condition compared to both the asynchronous (*p* = 0.002) and synchronous (*p* = 0.003) conditions. There is no significant difference between standard and anchoring conditions (*p* = 0.113), asynchronous conditions (*p* = 0.551), synchronous conditions (*p* = 0.462); between asynchronous and synchronous conditions (*p* = 0.749). This observation suggests that synchronized delays in feedback may hinder the operators' ability to effectively plan motor actions and manage time-based decision-making, consequently increasing brain activation. The anchoring condition, which provided immediate haptic feedback, appeared to promote a more efficient cognitive process, possibly by aiding in the temporal synchronization of motor actions and mitigating the disorienting effects of delayed visual feedback. It also highlights how the integration of haptic cues can support the cognitive processes involved in time perception, helping operators to maintain a coherent sense of timing despite the inherent delays in teleoperation.

#### 5.3.3 Primary motor cortex results

**Figure 12:**
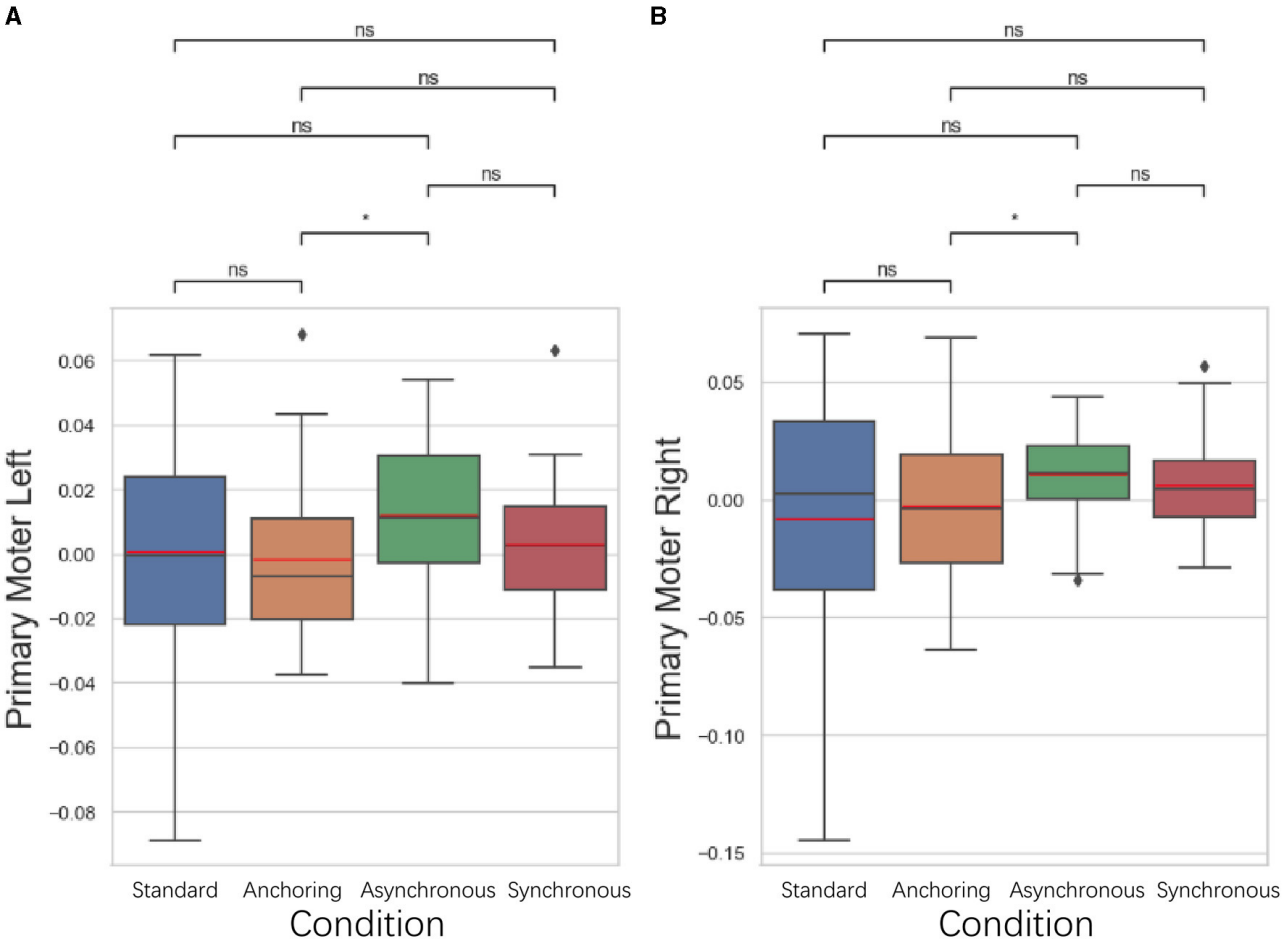
Statistical analysis results of oxyhemoglobin concentrations changes in **(A)** left primary motor cortex and **(B)** right primary motor cortex. ^*^Indicates statistically significant change (n.s., no significant difference, ^*^p < 0.05, ^**^p < 0.01, ^***^p < 0.001, ^****^p < 0.0001).

As illustrated in Figure 12, in the primary motor cortex, responsible for the execution of movements, anchoring condition demonstrated better performance compared to the asynchronous condition. The left primary motor cortex displayed a lower brain activation in the anchoring condition compared to the asynchronous condition (*p* = 0.037). There is no significant difference between standard and anchoring conditions (*p* = 0.539), asynchronous conditions (*p* = 0.180), synchronous conditions (*p* = 0.993); between anchoring condition and synchronous condition (*p* = 0.312); between asynchronous and synchronous conditions (*p* = 0.113). The right primary motor cortex also exhibited a lower brain activation in the anchoring condition compared to the asynchronous condition (*p* = 0.040). There is no significant difference between standard and anchoring conditions (*p* = 0.952), asynchronous conditions (*p* = 0.243), synchronous conditions (*p* = 0.517); between anchoring condition and synchronous condition (*p* = 0.204); between asynchronous and synchronous conditions (*p* = 0.243). This suggests that the stabilizing effect of immediate haptic feedback extends beyond planning and preparation, directly facilitating the actual motor execution. The reduction in brain activation observed in this region further supports the idea that the immediate feedback in the anchoring condition mitigates the challenges brought on by delayed visual feedback, enhancing motor execution efficiency.

#### 5.3.4 Premotor cortex results

**Figure 13:**
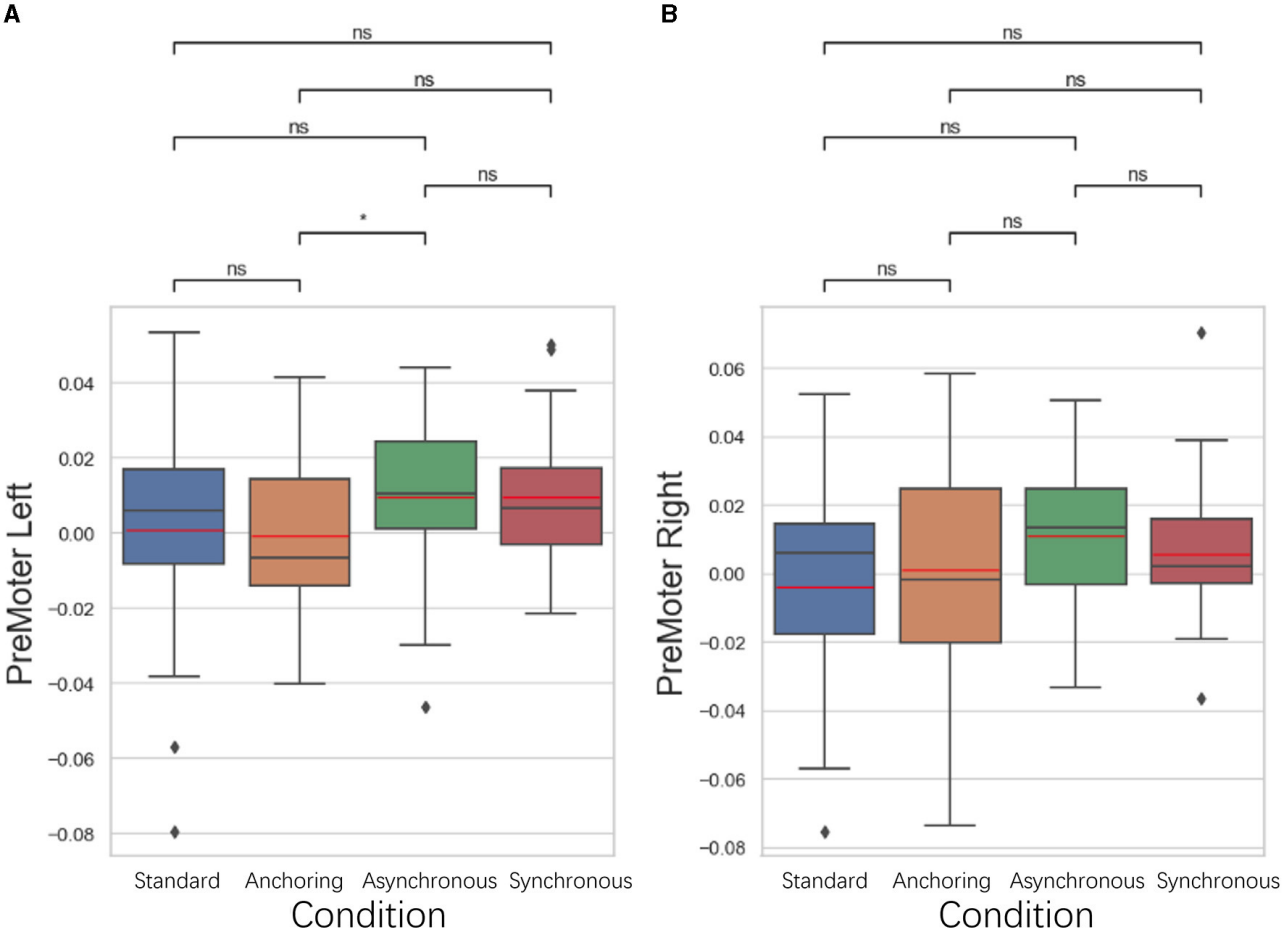
Statistical analysis results of oxyhemoglobin concentrations changes in **(A)** left premotor cortex and **(B)** right premotor cortex. ^*^Indicates statistically significant change (n.s., no significant difference, ^*^p < 0.05, ^**^p < 0.01, ^***^p < 0.001, ^****^p < 0.0001).

As illustrated in Figure 13, in the premotor cortex, focused on the organization and planning of movements, anchoring condition also showed better performance compared to the asynchronous condition. The left premotor cortex displayed a lower brain activation in the anchoring condition compared to the asynchronous condition (*p* = 0.039), suggesting that the immediate haptic feedback provided by the anchoring condition enhances the brain's ability to plan and prepare for movements. There is no significant difference between standard and anchoring conditions (*p* = 0.431), asynchronous conditions (*p* = 0.198), synchronous conditions (*p* = 0.462); between anchoring condition and synchronous condition (*p* = 0.058); between asynchronous and synchronous conditions (*p* = 0.550). For right premotor cortex, there is no significant difference between standard and anchoring conditions (*p* = 0.723), asynchronous conditions (*p* = 0.076), synchronous conditions (*p* = 0.634); between anchoring condition and asynchronous condition (*p* = 0.186) as well as synchronous condition (*p* = 0.452); between asynchronous and synchronous conditions (*p* = 0.257). This finding indicates that even in the presence of visual feedback delays, immediate haptic feedback can effectively support the cognitive processes involved in organizing motor actions, leading to more efficient motor planning and reduced brain activation.

Interestingly, despite the standard condition demonstrating better task performance, it was associated with a larger brain activity mean value across several cortical areas, including the prefrontal, right dorsolateral prefrontal cortex, and motor cortices. One possible explanation for this phenomenon is that: in the standard condition, without feedback delay, operators may adopt a strategy that emphasizes speed and accuracy, taking advantage of the immediacy of the system's responses. This could result in the utilization of more “cognitive energy” to maintain a high level of performance. The term “cognitive energy” here refers to the engagement and allocation of cognitive resources, such as attention, working memory, and executive functions, that are necessary to perform a task effectively.

Consequently, the fNIRS data indicated increased activity in the relevant brain regions, which might reflect this intensive cognitive engagement. This high level of activation could be interpreted positively as an indicator of the operators' active and focused state, enabling them to perform efficiently without delays. Conversely, in the anchoring condition and even more so in the asynchronous and synchronous conditions, the presence of feedback delays may require a shift in strategy. Operators had to first compensate for the “disruption” introduced by the delay, which could involve a more cautious approach, increased error-checking, or a reliance on alternative sensory feedback (like haptic cues in the anchoring condition). This shift could lead to a different pattern of brain activation, possibly a less intense one, as operators may spread their cognitive resources over a longer period due to the delay in feedback.

Therefore, the reduced activation in the anchoring condition compared to the standard condition might be due to a more distributed brain activation over time, rather than a concentrated burst of cognitive activity to immediately respond to feedback. This interpretation suggests that the high activation in the standard condition aimed at optimizing performance, whereas in the delayed conditions, cognitive efforts might be partly directed toward mitigating the negative impacts of delay.

It's important to note that these assumptions about the nature of cognitive activation are based on the observed data patterns and theoretical understanding of task demands. However, without direct evidence of the operators' strategies or subjective experiences, these interpretations remain speculative. Further research, perhaps incorporating qualitative data on operator strategies or additional quantitative measures, would be necessary to substantiate these hypotheses.

## 6 Discussion

Our human-subject experiment was designed to understand the neurofunctional implications of sensory manipulation in delayed robot teleoperation, yielded several insightful findings. Initially, when considering the neural data averaged across all phases of the experiment (pick-up, movement, and drop-off), no significant differences were observed among the four conditions: standard, anchoring, synchronous, and asynchronous. Nevertheless, a focused analysis on the pick-up phase (40 s) indicated differences among the four conditions. It suggests that the neurofunctional changes may have been event driven. And the pick-up phase represented a more difficult motor action, because the participants needed to move the robotic gripper to the center of the object, align well with the edge, and then grab the object, it did require more nuanced controls. While in contrast, the movement and the drop-off of the object on the target platform were comparably easier. As a result, we focused on the analysis of the pick-up phase.

In this phase, our initial hypothesis posited that the standard condition, characterized by simultaneous and delay-free visual and haptic feedback, would exhibit lower cognitive strain compared to conditions with delayed feedback. Contrary to our expectations, however, our findings did not reveal a significant reduction in cognitive strain in the standard condition relative to the anchoring condition. This observation suggests that even in the absence of sensory delays, the cognitive load required to manage multiple synchronous sensory inputs remains substantial.

One possible explanation for this phenomenon, as suggested by studies in the field of cognitive neuroscience, is that the higher activation observed in the standard condition may represent positive engagement with the task (Jansma et al., 2000). Engaging actively with multiple sensory channels might stimulate more extensive neural networks, reflecting a more involved and potentially enjoyable task experience. However, this higher activation could also signify cognitive strain. The need to constantly switch between visual and haptic feedback, as theorized by Alport et al. (1994), might place additional demands on cognitive resources, thereby increasing cortical activation. This scenario is consistent with the dual-task interference model, which suggests that managing multiple streams of sensory information can elevate cognitive load (Pashler, 1994).

Despite the cognitive demands being comparable across standard and anchoring conditions, the standard condition exhibited the best performance in terms of placement accuracy and time on task. This indicates that effective integration of synchronous sensory feedback, even at higher cognitive costs, may enhance performance. In contrast, in the anchoring condition, as visual feedback delay increases, participants may rely more heavily on haptic feedback and lessen their reliance on visual cues. This reduced sensory switching could lead to lower cortical activation but also results in poorer performance compared to the standard condition, where sensory integration is more balanced.

For anchoring, synchronous, and asynchronous conditions, the anchoring condition (immediate simulated haptic feedback with delayed visual cue) not only demonstrated improved motor performance but also showed a lower activation level in the anterior prefrontal cortex compared to both the synchronous and asynchronous conditions. This suggests a reduction in cognitive load. This aligns with the theory of cognitive load proposed by Sweller (1988), which posits that tasks with lower intrinsic cognitive demand result in lower cortical activation. By providing consistent haptic feedback, the anchoring condition may streamline the cognitive process, reducing the need for continuous cross-modal integration and error-checking that is more pronounced in conditions with asynchronous or no feedback. This reduction in cross-modal processing, as discussed in the multisensory integration literature (Stein and Stanford, 2008), may lead to a more efficient cognitive process with less prefrontal engagement.

Additionally, activation in the dorsolateral prefrontal cortex was similarly lower in the anchoring condition compared to both the synchronous and asynchronous conditions, reflecting a reduction in the cognitive demands of task management. This observation aligns with findings from Dockree et al. (2004), who noted that lower DLPFC activation correlates with reduced task-switching costs and more streamlined decision-making processes. Similarly, research by Paus (2001) suggests that decreased DLPFC activation during task performance could indicate more efficient cognitive control, particularly when participants become adept at utilizing consistent feedback to anticipate and adapt to task requirements. Such efficiency could explain the improved performance in motor tasks observed in the anchoring condition, as consistent haptic feedback may reduce the necessity for constant vigilance and adjustment prompted by varying sensory delays.

Furthermore, the anchoring condition led to reduced activation in the motor cortex compared to the asynchronous condition. The reduced activation in the motor cortex under the anchoring condition, significantly lower than in the asynchronous condition, reflects a more streamlined and efficient motor response. According to studies like Fitts and Posner (1967), as motor skills become more automated, the reliance on cognitive processes decreases, leading to reduced cortical activation. In the anchoring condition, the immediate haptic feedback might facilitate quicker motor learning and automation, thereby reducing the need for active motor planning and decision-making processes, typically associated with higher cortical activation. This efficiency could be attributed to a form of “sensorimotor tuning,” where the brain quickly adapts to the reliable haptic cues, optimizing motor outputs with less cognitive intervention (Wolpert et al., 2011).

However, it is important to note that there is no significant difference in motor cortex activation when comparing the anchoring condition with the standard and synchronous conditions. This observation suggests that the anchoring condition, while offering advantages over the asynchronous condition in terms of reduced motor cortex activation, exhibits similar activation levels to the standard condition. This similarity could be due to the consistent haptic feedback provided in both the anchoring and standard conditions, which may stabilize motor cortex activation despite variations in visual feedback delay. For the synchronous conditions, although both visual and haptic feedbacks are delayed, their simultaneous delay at equivalent levels could maintain a balance in sensory input, potentially preventing an increase in motor cortex activation. This synchronization might help preserve motor efficiency by ensuring that the discrepancies between sensory modalities do not exaggerate cognitive processing demands, thereby maintaining motor cortex activation at levels comparable to the standard and anchoring conditions.

These findings underscore the complex interplay between sensory feedback, motor coordination, and cognitive processing in teleoperation. They highlight that while reducing cognitive load through fewer sensory switches might decrease cortical activation, it does not necessarily translate to improved task performance. Future research should aim to disentangle these aspects further, possibly using subjective measures of task engagement and cognitive strain in conjunction with neuroimaging data. Additionally, exploring variations in task complexity and sensory feedback modalities could provide deeper insights into optimizing teleoperated systems for both performance efficiency and user experience.

## 7 Conclusions

This research is driven by the motivation to understand the neurofunctional implications of sensory manipulation in delayed robot teleoperation, a field that, despite its technological advancements, still hindered by the challenges of communication delays. The primary goal of this research is to fill a critical knowledge gap: the lack of neurofunctional evidence regarding the impact of simulated, synthetic haptic feedback on neural functions, especially those related to time perception and motor coordination. Delays in teleoperation can significantly affect performance, but the underlying neural dynamics, particularly in the context of sensory augmentation, remained largely unexplored. By focusing on these aspects, our study aims to provide insights that could lead to more intuitive and effective teleoperated systems, especially in applications demanding precision and timeliness.

Our human-subject experiment, involving different conditions of sensory feedback in teleoperation, revealed that the anchoring condition, with immediate simulated haptic feedback, not only improved motor performance but also regulated the activation levels of key brain regions such as the DLPFC and the APFC. This finding is significant as it suggests that providing real-time synthetic force feedback can reduce the cognitive and motor challenges posed by delayed teleoperation, particularly in the more demanding pick-up phase of the task. The reduction in DLPFC and APFC activation under the anchoring condition points toward a potential decrease in cognitive load and enhanced motor coordination. These results contribute to the understanding of how synthetic sensory feedback can be optimized to improve teleoperated task performance, providing a foundation for future technological developments in this area.

While our findings are promising, they are not without limitations. The study's scope was confined to a controlled experimental setting, which might not fully capture the complexities of real-world teleoperation scenarios. Additionally, the focus on specific brain regions, though insightful, does not encompass the entire spectrum of neural processes involved in teleoperation. Future research should aim to replicate these findings in more varied and dynamic settings to verify their applicability in real-world applications. Furthermore, exploring other forms of sensory manipulation and their neurofunctional impacts, as well as investigating the long-term effects of such interventions on skill acquisition and adaptation in teleoperation, would be beneficial. These future agenda items could provide deeper insights into the neural mechanisms underlying teleoperated systems, guiding the development of more responsive, efficient, and user-friendly teleoperation technologies.

## Data availability statement

The datasets presented in this study can be found in online repositories. The names of the repository/repositories and accession number(s) can be found at: https://www.dropbox.com/scl/fo/4qcvc4v3nxhc9in0oi6bo/h?rlkey=15eds1e7on4vjkstannruok5r&dl=0.

## Ethics statement

The studies involving humans were approved by University of Florida Institutional Review Board. The studies were conducted in accordance with the local legislation and institutional requirements. The participants provided their written informed consent to participate in this study.

## Author contributions

TZ: Formal analysis, Methodology, Software, Writing – original draft. YY: Formal analysis, Methodology, Visualization, Writing – review & editing. QZ: Formal analysis, Methodology, Software, Validation, Writing – review & editing. WV: Data curation, Investigation, Methodology, Software, Validation, Visualization, Writing – review & editing. JD: Conceptualization, Funding acquisition, Methodology, Project administration, Supervision, Writing – review & editing.
